# Exploiting β‐Lactams‐Induced Lysis and DNA Fragmentation for Rapid Molecular Antimicrobial Susceptibility Testing of *Neisseria Gonorrhoeae* via Dual‐Digital PCR

**DOI:** 10.1002/advs.202405272

**Published:** 2024-10-18

**Authors:** Jiumei Hu, Liben Chen, Pengfei Zhang, Fan‐En Chen, Hui Li, Kuangwen Hsieh, Sixuan Li, Johan H. Melendez, Tza‐Huei Wang

**Affiliations:** ^1^ Department of Mechanical Engineering Johns Hopkins University Baltimore MD 21218 USA; ^2^ Department of Biomedical Engineering Johns Hopkins School of Medicine Baltimore MD 21205 USA; ^3^ Division of Infectious Diseases Department of Medicine Johns Hopkins University School of Medicine Baltimore MD 21205 USA; ^4^ Institute for NanoBiotechnology Johns Hopkins University Baltimore MD 21218 USA

**Keywords:** antimicrobial resistance, bacterial lysis, DNA fragmentation, microfluidic dual‐digital PCR, *Nesseria gonorrhoeae (N. gonorrheoae)*

## Abstract

The evolution of antimicrobial resistance (AMR) presents substantial challenges to global medical health systems. *Neisseria gonorrhoeae* (*N. gonorrhoeae*), in particular, has developed resistance to all currently available antimicrobials. Addressing this issue necessitates not only discovering new antimicrobials but also deepening the understanding of bacterial responses to these agents, which can lead to new markers for rapid antimicrobial susceptibility testing (AST). Such advancements can enhance treatment outcomes and promote antimicrobial stewardship. In this study, single‐cell techniques, including live‐cell imaging, flow cytometry, and digital polymerase chain reaction (PCR) are utilized, to investigate the lysis dynamics and molecular features of *N. gonorrhoeae* upon exposure to β‐lactam antimicrobials. Distinct patterns of bacterial lysis and DNA fragmentation are uncovered in susceptible strains. Leveraging these discoveries, a microfluidic dual‐digital PCR approach that combines single‐cell and single‐molecule analyses, facilitate rapid and efficient phenotypic molecular AST for *N. gonorrhoeae* against β‐lactams is developed. This proof‐of‐concept validation demonstrates the effectiveness of the method in accessing antimicrobial susceptibility across a range of bacterial strains, contributing valuable insights for advancing the battle against AMR.

## Graphical abstract

Utilizing DNA fragmentation and abrupt bacterial lysis induced by β‐lactam antimicrobials, a microfluidic dual‐digital PCR that integrates single‐cell and single‐molecule analyses for rapid phenotypic molecular antimicrobial susceptibility testing of *Neisseria gonorrhoeae* to β‐lactams is developed.

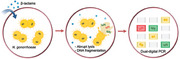

## 1 Introduction

Antimicrobial resistance (AMR) poses an escalating threat to public health infrastructure that is heavily reliant on antimicrobials for managing bacterial infections. With estimates suggesting that AMR was responsible for over 1.27 million deaths worldwide in 2019, it stands as a leading cause of mortality globally.^[^ ^1^ ^]^ This widespread issue is largely driven by the overuse or misuse of antimicrobials,^[^ ^2^ ^]^ leading to bacteria developing and evolving various mechanisms to withstand antimicrobial effects. The current state in the fight against AMR is precarious, as certain bacteria have already developed or acquired resistance to all available antimicrobials, thus becoming superbugs that defy current treatments.^[^ ^3^, ^4^ ^]^ Projections indicate that without intervention, AMR could result in 10 million deaths annually by 2050, potentially surpassing the mortality rate of cancer.^[^ ^5^ ^]^

*Neisseria gonorrhoeae* (*N. gonorrhoeae*), the causative bacterium of gonorrhea, has become a major public health concern.^[^ ^6^ ^]^ Over the years, it has developed resistance to numerous antimicrobials, including ceftriaxone (CEF), the last remaining empirical treatment for *N. gonorrhoeae* infections.^[^ ^7^ ^]^ Untreated gonorrhea can result in serious consequences beyond the immediate impacts of the infection itself, such as pelvic inflammatory disease, infertility in women, increased risk of HIV acquisition, and various neonatal diseases via mother‐to‐child transmission.^[^ ^8^ ^]^ While the development of new antimicrobials or therapies is crucial, the prolonged timeline, often spanning a decade or longer, means our drug discovery efforts are outpaced by the rapid evolution of AMR. Moreover, despite extensive antimicrobial use, our understanding of their impact on bacterial physiology and morphology remains incomplete.^[^ ^9^, ^10^, ^11^ ^]^ Thus, a more in‐depth exploration of bacterial responses to antimicrobials is critical for developing new treatments and diagnostic biomarkers to mitigate resistance.^[^ ^10^ ^]^

Current research on AMR in *N. gonorrhoeae* has mainly concentrated on how biomolecules, especially DNA and proteins, adapt to antimicrobials.^[^ ^12^, ^13^ ^]^ Studies employing genome‐wide and proteome‐wide analyses, such as next‐generation sequencing and mass spectrometry, have greatly enhanced our understanding of bacterial resistance mechanisms.^[^ ^14^, ^15^ ^]^ meanwhile, these studies have led to the development of novel strategies to fight against AMR. For instance, the identification of genetic markers of AMR has enabled the development of rapid testing methods for antimicrobial susceptibility assessment, utilizing techniques like nucleic acid amplification tests (NAATs).^[^ ^16^, ^17^, ^18^, ^19^ ^]^ These rapid tests are crucial in managing AMR, allowing healthcare providers to promptly prescribe antimicrobials, thus reducing the misuse of broad‐spectrum antimicrobials – a primary driver of AMR. However, it is important to recognize the limitations of genetic‐based methods, as they may not always accurately predict the actual resistance phenotype due to the complex nature of bacterial resistance mechanisms.^[^ ^20^, ^21^, ^22^ ^]^ Furthermore, other research on AMR, such as bacterial physiological and morphological changes under antimicrobial exposure, while documented in other bacterial species like *Escherichia coli* (*E. coli*),^[^ ^23^, ^24^, ^25^ ^]^ remain largely unexplored in *N. gonorrhoeae*.

In this study, we examined the responses of *N. gonorrhoeae* to β‐lactam antimicrobials, the primary treatment to this infection, focusing on lysis dynamics and molecular characteristics under the stress of these antimicrobials. Employing single‐cell techniques including live‐cell imaging, flow cytometry, and digital polymerase chain reaction (PCR), we observed notable cellular responses. Remarkably, a considerable proportion of β‐lactam‐susceptible cells displayed the biochemical hallmark of apoptosis, specifically DNA fragmentation, after one hour of antimicrobial exposure. Furthermore, we observed distinct lysis phenotypes, with a subset of cells undergoing abrupt lysis and releasing fragmented DNA immediately following treatment. These findings prompted the development of a microfluidic dual‐digital PCR method for simultaneously quantifying bacterial cell counts and extracellular free DNA fragments, facilitating a rapid phenotypic molecular antimicrobial susceptibility testing (AST) for *N. gonorrhoeae* against β‐lactam antimicrobials. Applying this technique, we evaluated the susceptibility of clinical isolates to two β‐lactam antimicrobials, penicillin (PEN) and CEF. Our results showed a strong correlation between the outcomes of our rapid phenotypic molecular AST, conducted after just one hour of antimicrobial exposure, and the traditional, more time‐consuming culture‐based AST methods. These results suggest an effective new avenue for developing rapid phenotypic AST techniques for *N. gonorrhoeae*, offering significant potential to enhance global health response strategies.

## 2 Results

### 2.1 PEN Inhibits Cell Proliferation and Induces Distinct Bacterial Lysis Phenotypes

**Figure 1:**
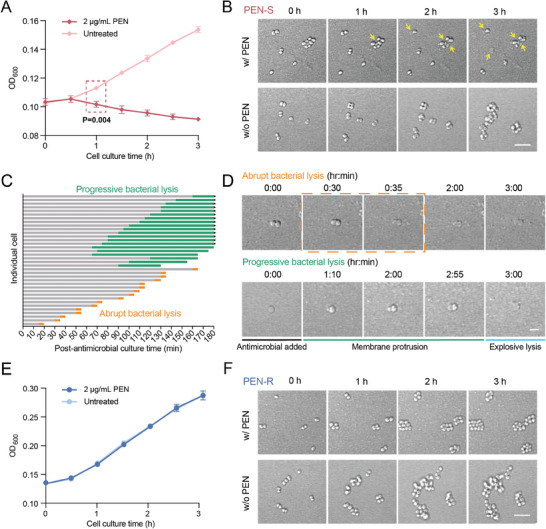
Dynamics of bacterial cell lysis. A) Representative optical density changes over time of PEN‐susceptible strain (ATCC 43069). Student *t*‐test was performed on the data obtained from treated and untreated cells after 1‐hour PEN exposure (*n* = 3). B) ATCC 43069 was cultured in GW medium supplemented with 2 µg mL^−1^ PEN, alongside an untreated control. The bacteria cultures were then imaged under the Zeiss LSM780 confocal microscope. Yellow arrows represent the cells that were lysed within the 3‐hour timeframe. Scale bar: 5 µm. C) Bar plot showing the two bacterial lysis phenotypes observed under the microscope in 3 h, with images captured every 5 min. Each bar represents the status of one cell post‐PEN exposure. The grey segments depict cell status without discernable morphological changes. Orange segments indicate the onset and completion of abrupt lysis, which can complete under 5 min. Green segments show the start and end of progressive lysis. Bars marked with black arrows show cells where lysis was incomplete within the 3‐hour observation timeframe. D) Images showing the dynamics of the two bacterial lysis phenotypes. Scale bar: 2 µm. E) Representative optical density changes over time of PEN‐resistant strain (clinical isolate 16‐04‐11) (*n* = 3). F) Live‐cell microscopic images showed that the growth of the PEN‐resistant strain was not affected by antimicrobial exposure. Scale bar: 5 µm.

β‐lactam antimicrobials inhibit the synthesis of peptidoglycan in bacterial cell wall by covalently binding to penicillin‐binding proteins, major synthases participating in peptidoglycan assembly. Although the inhibition on peptidoglycan synthesis is generally thought to induce bacterial lysis, the lysis patterns can vary substantially among different species and the underlying mechanisms remain incompletely understood.^[^ ^26^ ^]^ To evaluate the impact of β‐lactam antimicrobials on *N. gonorrhoeae*, we first performed real‐time optical density (OD) measurements at the cell population level using an ATCC (American Type Culture Collection) bacterial strain susceptible to PEN. Our results showed that PEN induced *N. gonorrhoeae* lysis, as indicated by a decrease in OD_600_ overtime, while the untreated cells sustained undisturbed proliferation (**Figure** **1**A).

To gain a deeper understanding about the cellular dynamics during β‐lactam‐induced bacterial lysis, we further conducted live‐cell imaging under a confocal microscope at the single‐cell level. Consistent with the OD measurement results, cells under PEN treatment eventually stop proliferation, concomitant with induced bacterial lysis overtime (Figure 1B, top). Within the PEN‐treated cell population, we observed highly diverse responses among individual bacteria to antimicrobial treatment, primarily on the speed they reacted to the antimicrobial and the distinct lysis phenotypes. Notably, two lysis phenotypes were identified including abrupt bacterial lysis and progressive bacterial lysis that occur at different time scales (Figure 1C). The abrupt bacterial lysis features a sudden loss of cell integrity that lasts less than 5 min, which unfolds across various time points, with the earliest instances occurring within 20 min of antimicrobial exposure. Comparatively, the progressive bacterial lysis is a dynamic process with two recognizable phases including membrane protrusion and explosive cell lysis, which overall initiates at a relatively later stage and typically takes tens of minutes to hours to complete (Figure 1D, Video S1, Supporting Information). In contrast, the untreated cells exhibited normal proliferation, with cell doubling observed at varied time points for individual cells owning to asynchronous bacterial growth (Figure 1B, bottom). Collectively, the cell number decrease in treated cells after bacterial lysis and increase in untreated cells due to bacterial growth revealed an increasingly evident difference in cell quantities between the two groups, in which a statistically significant difference (*P* < 0.05, student *t*‐test, Figure 1A) was consistently observed even after only one hour of PEN exposure. Meanwhile, we conducted identical experiments on a clinically isolated *N. gonorrhoeae* strain that is resistant to PEN. As anticipated, PEN treatment showed no discernible impact on this strain, as revealed by both the OD measurement (Figure 1E) and live‐cell imaging results (Figure 1F, Video S2, Supporting Information).

### 2.2 PEN Induces DNA Fragmentation during Acute Exposure

**Figure 2:**
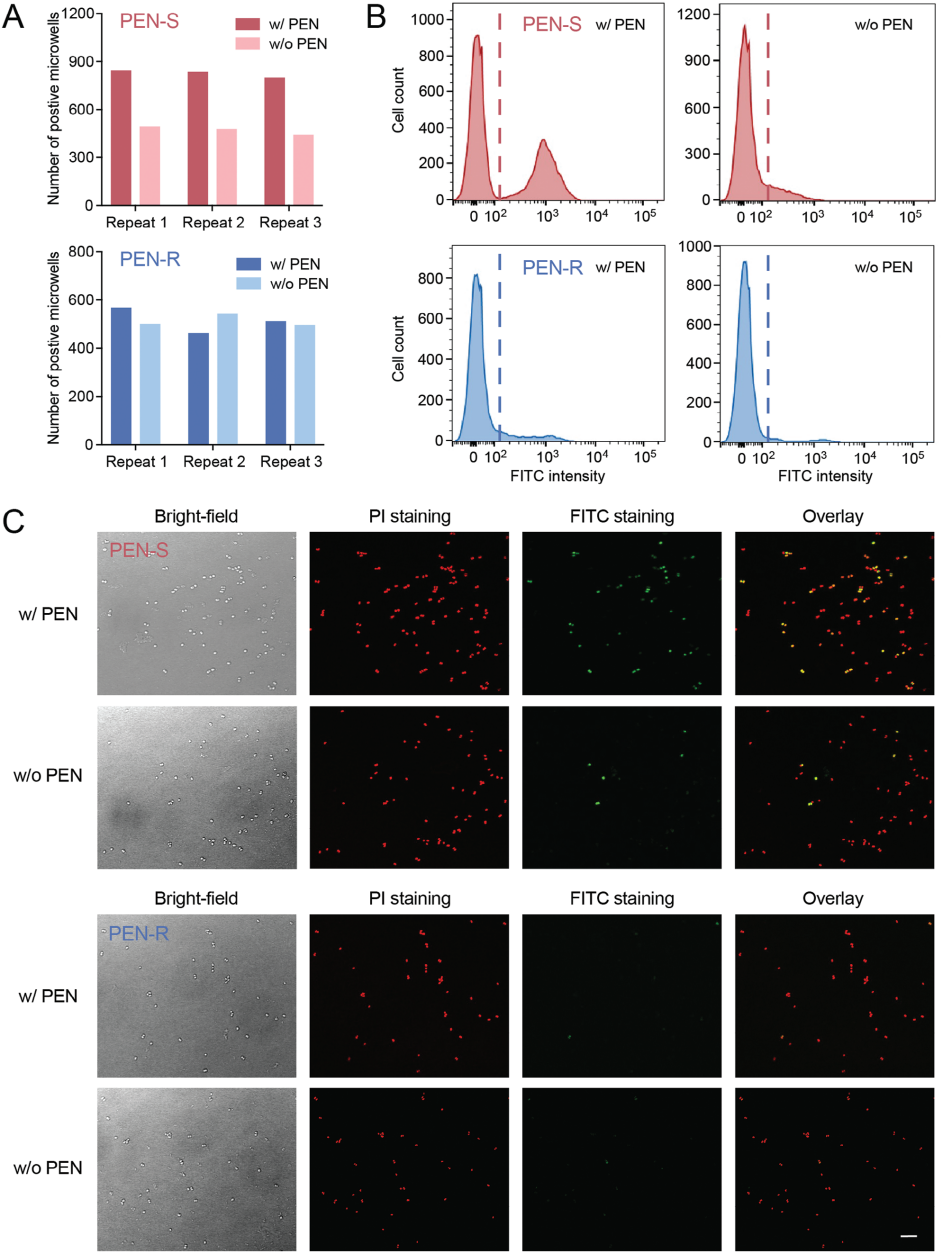
PEN induces DNA fragmentation in susceptible *N. gonorrhoeae*. A) ATCC 43069 and clinical isolate 16‐04‐11 strains were exposed to 2 µg mL^−1^ PEN for an hour, along with an untreated control, respectively. Around 500 cells from each sample were digitalized and detected on our microfluidic chip via TaqMan digital PCR targeting the *opa* gene. B) Histograms showing the percentage of TUNEL‐positive cells. Susceptible cells treated with PEN for one hour underwent more DNA fragmentation compared with the untreated sample, while such a difference was not observed between PEN‐treated and untreated resistant cells. C) Stained *N. gonorrhoeae* cells were also imaged on a ZEISS LSM780 confocal microscope, revealing similar results as observed on flow cytometry. Scale bar: 10 µm.

To more accurately quantify the cell number difference after PEN exposure as revealed in the OD measurement and live‐cell imaging results above, we utilized the digital PCR with a customized microfluidic array^[^ ^27^ ^]^ for single cell quantification. This approach involved digitalizing individual cells into discrete microwells followed by PCR amplification targeting the *N. gonorrhoeae‐*specific *opa* gene. Our results showed comparable number of positive microwells between antimicrobial‐treated and untreated cells for the PEN‐resistant *N. gonorrhoeae*. However, in the PEN‐susceptible *N. gonorrhoeae*, the outcome diverged significantly from our initial expectation, as the antimicrobial‐treated cells exhibited a much higher number of positive microwells compared with the untreated cells (**Figure** **2**A; Figure S1, Supporting Information). This unexpected observation might be attributed to DNA fragmentation in PEN‐susceptible *N. gonorrhoeae* cells upon PEN exposure. As a result, the multiple copies of the *opa* gene within each cell were released into the cell culture medium following bacterial lysis, leading to an increased number of *opa*‐positive events on the digital PCR microarray.

To verify this hypothesis, we performed the standard DNA fragmentation assay – the terminal deoxynucleotidyl transferase dUTP nick‐end labeling (TUNEL) using flow cytometry on antimicrobial‐treated and untreated *N. gonorrhoeae* cells. TUNEL assay utilizes Fluorescein isothiocyanate‐conjugated deoxy uridine triphosphate (FITC‐dUTP) to directly label the 3′‐hydroxyl ends of DNA breaks so that cells with fragmented DNA can emit stronger fluorescence than cells with intact DNA. Meanwhile, Propidium Iodide (PI) is adopted to stain the total DNA in cells and exclude any non‐cell particles or debris from analysis. A TUNEL‐positive signal is indicated by the coincidence of both FITC and PI fluorescence. For PEN‐susceptible *N. gonorrhoeae*, our results showed that a significantly higher percentage of TUNEL‐positive cells was observed in antimicrobial‐treated cells than untreated cells, indicating the occurrence of DNA fragmentation induced by antimicrobial exposure. In comparison, there was no significant difference in TUNEL results from resistant *N. gonorrhoeae* cells (Figure 2B; Figure S2, Supporting Information). Additionally, individual cells were also imaged under a microscope, showing the highly efficient PI staining for bacteria identification as well as the successful determination of cells with DNA fragmentation (Figure 2C). Collectively, these results suggest that PEN induces DNA fragmentation in the susceptible *N. gonorrhoeae*.

### 2.3 Dual‐Digital PCR using Two‐Color Coincidence Analysis for Simultaneous Quantification of Cells and Extracellular DNA Fragments

Based on our findings, we reason that the phenomena of abrupt cell lysis and DNA fragmentation in antimicrobials‐susceptible cells could serve as a basis for a rapid antimicrobial susceptibility assessment method for *N. gonorrhoeae* against β‐lactam antimicrobials, utilizing the quantities of intact cells and cell‐free DNA fragments as surrogate markers. However, the pronounced tendency of *N. gonorrhoeae* cells for lysis under alkaline conditions,^[^ ^28^ ^]^ including those introduced by PCR reagents as demonstrated in Figure S3 (Supporting Information), may compromise this approach. In typical digital PCR protocols, where cell samples are mixed with PCR reagents before being loaded onto the digital PCR chip, cell disruption could occur, complicating the use of cell lysis and DNA fragmentation as reliable markers for AST in *N. gonorrhoeae*. To circumvent this, we utilized a previously developed microfluidic digital array chip,^[^ ^27^ ^]^ engineered for performing multi‐step digital assay, to facilitate sequential loading of cell samples and PCR reagents (Figure S4, Supporting Information), effectively avoiding the issue.

**Figure 3:**
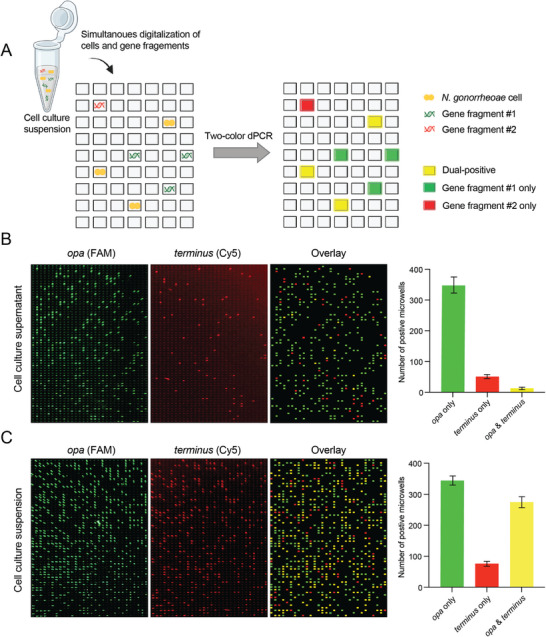
Dual‐digital PCR using two‐color coincidence analysis for the co‐detection of extracellular free DNA and *N. gonorrhoeae* cells. A) Illustration showing the concept of utilizing coincidence analysis for simultaneous quantification of extracellular free DNA fragments and cells in cell culture suspension via a two‐color digital PCR. B) Cell culture supernatant obtained from the one‐hour PEN‐treated susceptible *N. gonorrhoeae* cell sample (ATCC 43069) was tested on our chip via two‐color TaqMan PCR targeting *opa* (FAM) and *terminus* (Cy5) genes. The bar plot on the right showed the number of microwells containing only *opa* or *terminus* genes, or both genes, showing the reliable detection of DNA fragments. Error bars represent data obtained from three repeated experiments. C) Cell suspension from the same sample was further tested on our chip, showing the capability of our method to detect extracellular free DNA and cells simultaneously. Error bars on the bar plot represent data obtained from three repeated experiments.

By utilizing this microfluidic digital array, we are able to isolate individual cells and DNA fragments within separate microwells for PCR amplification. This allows for the implementation of two‐color coincidence analysis to simultaneously measure two genomic loci of *N. gonorrhoeae*: the *opa* gene, detectable in the FAM channel, and a genomic segment near the bacterial chromosome end, known as the *terminus*, detectable in the Cy5 channel. The selection of the *terminus* locus, due to its considerate spatial separation from the *opa* gene on the bacterial genome, ensures effective segregation of the two gene fragments upon bacterial DNA fragmentation. We anticipate that a microwell containing a cell will co‐amplify two genomic loci on the bacterial genome, leading to the generation of two distinct fluorescence colors. In contrast, DNA fragmentation results in the separation of distinct loci into individual fragments, each isolated in microwells as independent units. Consequently, a microwell containing DNA fragments will amplify only the specific locus present, resulting in a singular fluorescence color (**Figure** **3**A).

To confirm the efficacy of our dual‐digital PCR assay, we first verified the high PCR amplification efficiencies for *opa* and *terminus* through a single reaction using bulk real‐time PCR (Figure S5, Supporting Information). We then proceeded with separate experiments using a one‐hour PEN‐treated susceptible *N. gonorrhoeae* cell culture sample to validate the capacity of our approach in detecting intact cells and extracellular DNA fragments. First, we digitalized the cell culture supernatant, which contains only extracellular DNA fragments, and then measured it using two‐color coincidence analysis. The results consistently showed a substantial number of microwells exhibiting either *opa* or *terminus* positivity, with only a minimal number of microwells showing positivity for both (Figure 3B). Notably, the number of *opa*‐positive microwells significantly exceeded that of *terminus*‐positive ones, reflecting the genomic structure of *N. gonorrhoeae*, where *opa* is a multi‐copy gene, while *terminus* is a single‐copy segment. This result validates our observations related to abrupt cell lysis and DNA fragmentation, resulting in the release of extracellular DNA following a short period of antimicrobial exposure.

Second, we digitalized the entire cell culture suspension, which includes *N. gonorrhoeae* cells and extracellular DNA fragments, and subjected it to the two‐color digital PCR analysis. The analysis revealed a high incidence of microwells positive for both *opa* and *terminus*. The quantity of the dual‐positive wells was consistent with the initial cell input of ≈250 CFU, indicating accurate quantification of intact cells. Additionally, the amount of extracellular *opa* or *terminus* DNA fragments was comparable to those measured in the cell culture supernatant (Figure 3C), demonstrating the method's effectiveness in detecting and quantifying both cellular and extracellular DNA components.

### 2.4 Streamlined Workflow for Performing *N. Gonorrhoeae* AST to PEN

Utilizing the dual‐digital PCR method, we developed a streamlined phenotypic molecular AST workflow for the assessment of *N. gonorrhoeae* susceptibility to β‐lactam antimicrobials. Our procedure begins with incubating *N. gonorrhoeae* cells in GW medium with antimicrobials for an hour, alongside a control group without antimicrobials. Subsequently, both cell samples are loaded into two adjacent modules on our digital PCR array, followed by the addition of PCR buffer through the two‐step loading scheme (Figure S4, Supporting Information). After performing a two‐color coincidence analysis, we quantify the microwells containing either the *opa* gene or the *terminus* locus, which signify the presence of the extracellular free DNA fragments (*N_DNA_*). Concurrently, we determine the number of microwells exhibiting amplification of both loci, which correspond to intact *N. gonorrhoeae* cells (*N*_cell_). The ratio of *N*_DNA_ to *N*_cell_, termed as DNA‐to‐cell ratio (*DCR*), is utilized to assess the antimicrobial susceptibility of an *N. gonorrhoeae* strain by comparing the *DCR* ratios from the antimicrobial‐treated and ‐untreated groups, thereby deriving a susceptibility score *S_s_*,

(1) $$S_{S}=\frac{\mathrm{DC}R_{\mathrm{treated}}}{\mathrm{DC}R_{\mathrm{untreated}}}$$

**Figure 4:**
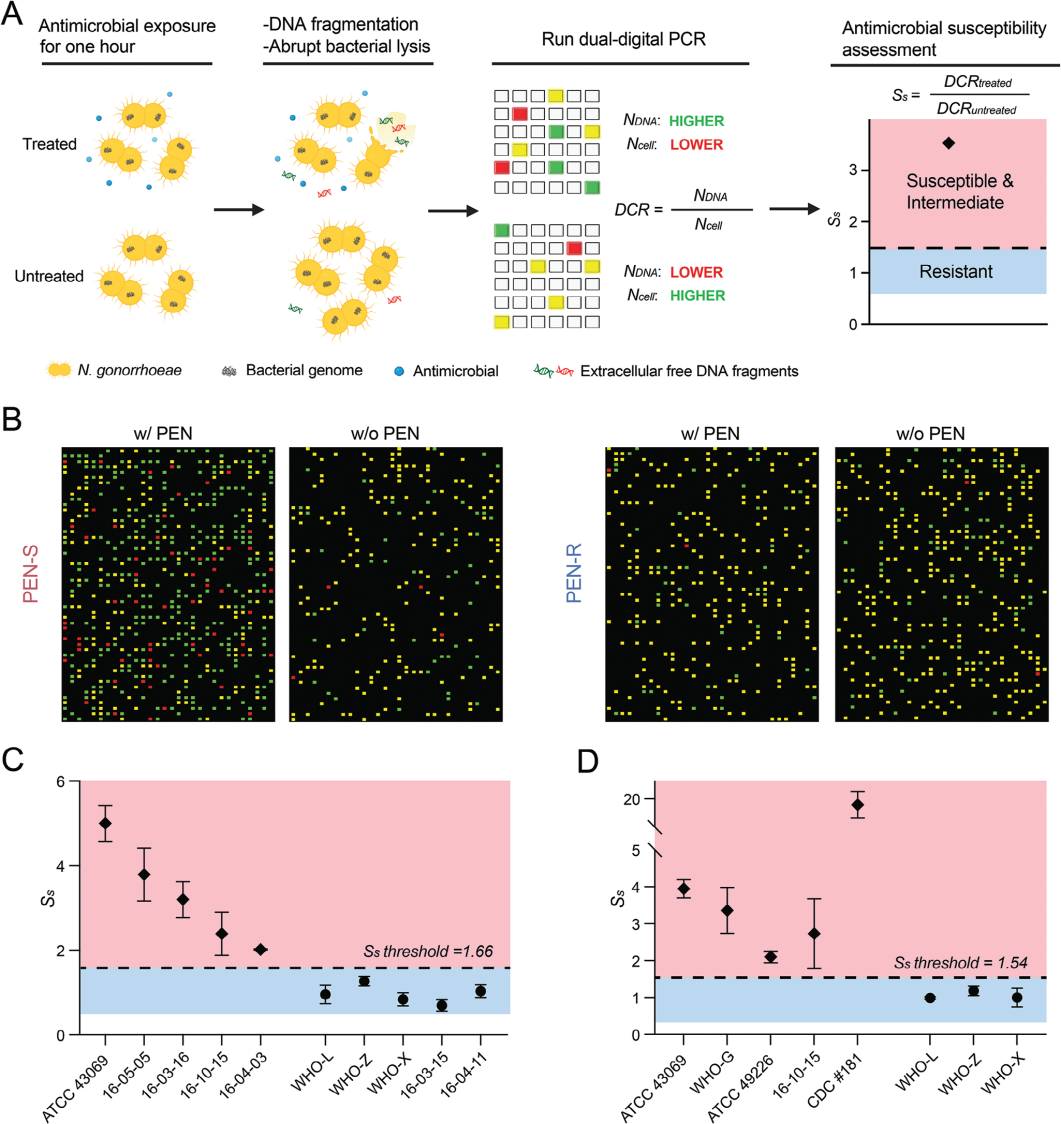
*N. gonorrhoeae* AST to PEN and CEF via dual‐digital PCR. A) The workflow of our approach shown for *N. gonorrhoeae* susceptible to β‐lactam antimicrobials. The cell sample is divided into two parts: one treated with antimicrobials, while the other serves as an untreated control. After an hour of antimicrobial exposure, cell growth is inhibited, along with the occurrence of DNA fragmentation as well as the abrupt lysis to release the fragmented DNA. Simultaneously quantifying DNA fragments and cells in the microwell array allows us to calculate the ratio of free DNA to cell number for each sample (DCR). Treated cells exhibit a higher ratio, while untreated cells show a lower ratio. Further taking the ratio of these two DCRs establishes the criterion (Ss) for our AST approach, where susceptible and intermediate cell samples have a Ss value greater than one, and resistant cell samples have a value close to one. The black rhombus indicates the detected susceptibility of the strain, showing a Ss value greater than one. B) Representative AST results for the PEN‐susceptible cells (ATCC 43069) and PEN‐resistant cells (clinical isolate 16‐04‐11). C) Different strains were tested, and their susceptibilities to PEN were correctly determined using our approach with an Ss threshold of 1.66. D) The susceptibility of multiple strains to CEF was also tested using our approach, showing reliable differentiation between susceptible and resistant cell groups with a Ss threshold of 1.54. Each dataset contains at least two replicates.

In cell deemed susceptible or intermediate, treatment with antimicrobials is anticipated to increase the quantity of cell‐free DNA and decrease the cell count relative to untreated samples, resulting in a *S_s_* value generally above one. On the contrary, in resistant cells unaffected by the antimicrobials, the *S_s_* value is expected to hover around one (**Figure** **4**A).

We initially tested the efficacy of our AST method using susceptible (ATCC 43069) and resistant (clinical isolate 16‐04‐11) strains. As shown in Figure 4B, cells susceptible to PEN showed notable differences in cell counts and amounts of extracellular free DNA following antimicrobial treatment, unlike the untreated cells. Conversely, the resistant cells showed comparable results between the antimicrobial‐treated and untreated groups. To further corroborate the effectiveness of our method, we performed AST on ten clinical isolates, including two PEN‐susceptible, three PEN‐intermediate, and five PEN‐resistant *N. gonorrhoeae* strains (Table S1, Supporting Information). The tests were conducted at a PEN concentration of 2 µg mL^−1^, the breakpoint value established by the Clinical and Laboratory Standards Institute (CLSI). To categorize these *N. gonorrhoeae* strains, we established an *S_s_* threshold by calculating the mean plus three times the standard deviation (mean + 3SD) of the *S_s_* values obtained from all tested resistant strains. The results showed that all the PEN‐non‐resistant strains (susceptible and intermediate) were clearly differentiable from the resistant strains, indicating the effectiveness of our approach for antimicrobial susceptible assessment of *N. gonorrhoeae* to PEN (Figure 4C).

It's noteworthy that traces of extracellular free DNA were observed in samples from both untreated cells and antimicrobial‐treated resistant cells, likely due to autolysis during the culturing process (Video S3, Supporting Information). This phenomenon could also result in DNA fragmentation, as indicated by the TUNEL assay result (Figure 2B). The presence of such background extracellular free DNA might affect the accuracy of bacterial antimicrobial susceptibility assessment. Nevertheless, our approach effectively addressed this issue by combining the measurement of free DNA with cell quantification for conducting AST. As a comparison, we evaluated the performances of approaches relying solely on either extracellular free DNA or cell counts as the exclusive metric for differentiating PEN‐non‐resistant from PEN‐resistant strains. For each approach, we established threshold values in a similar way as the *S_s_* threshold, defined as the mean + 3SD of extracellular free DNA amount ratio (treated/untreated) or cell count ratio (untreated/treated) from all tested resistant strains. Our results showed that while extracellular free DNA effectively differentiated most non‐resistant strains (4 out of 5) from all resistant strains, it was insufficient for differentiating strains with only a slight difference in MIC from resistant strains (Figure S6A, Supporting Information). Using cell counts only proved highly unreliable for distinguishing different *N. gonorrhoeae* strains (Figure S6B, Supporting Information). Altogether, these results underscore the effectiveness of our dual‐digital PCR approach where both metrics are utilized simultaneously.

### 2.5 Antimicrobial Susceptibility Assessment of *N. Gonorrhoeae* to CEF

Our molecular AST approach was extended to include testing with CEF, the last remaining empirical first‐line treatment of gonorrhea. We subjected both a susceptible strain (ATCC 43069) and a resistant strain (WHO‐X) to CEF treatment at a concentration of 0.25 µg mL^−1^ for an hour and then applied our molecular AST on the resulting cell culture suspension. The resistant strain showed an expected *S_s_* value of around one, while the susceptible strain did not show an increased score. By extending the antimicrobial treatment time to two hours, we were able to accurately differentiate between the strains, as demonstrated in Figure S7 (Supporting Information).

Recognizing the critical influence of antimicrobial concentration in bacterial response, we investigated the possibility to expedite the process of molecular AST by increasing the concentration of the antimicrobial treatment, aiming to reduce the duration of CEF exposure to be within an hour. To this end, we tested a variety of *N. gonorrhoeae* strains, both susceptible and resistant, with different MICs (Table S1, Supporting Information). Using OD measurements, we evaluated the response of these strains to varied concentrations of CEF ranging from 0.25 to 20 µg mL^−1^ over the course of an hour. Our observations revealed marked growth inhibition in susceptible strains with increasing CEF concentrations, whereas resistant strains did not exhibit significant growth inhibition until the CEF concentration exceeded 5 µg mL^−1^ (Figure S8, Supporting Information).

By applying a 5 µg mL^−1^ concentration of CEF, we performed our molecular AST on the aforementioned strains (ATCC 43069 and WHO‐X) with only one hour of antimicrobial exposure. This approach distinctly identified the susceptible strain from the resistant one, demonstrated by a notable increase in extracellular free DNA and a decrease in cell counts for the CEF‐treated susceptible strain (Figure S9, Supporting Information). We then extended our assessment to other susceptible and resistant strains under the same conditions. This method effectively distinguished the susceptibility scores obtained from the two groups, setting a threshold value at 1.54, thereby validating the effectiveness of our approach in assessing antimicrobial susceptibility across all strains tested (Figure 4D).

## 3 Discussion

In this study, we noted two physiological responses in *N. gonorrhoeae* following exposure to β‐lactam antimicrobials. First, susceptible strains exhibit two distinct patterns of cell lysis: abrupt and progressive bacterial destruction. Furthermore, we discovered DNA fragmentation in these bacteria post‐exposure to β‐lactam antimicrobials. Leveraging these insights, we developed a microfluidic dual‐digital PCR assay capable of concurrently quantifying bacterial cells and extracellular free DNA fragments. This innovative approach facilitates a phenotypic molecular AST method for assessing the susceptibility of *N. gonorrhoeae* to β‐lactam antimicrobials, including PEN and CEF, efficiently within an hour of antimicrobial exposure.

The rapid lysis of bacterial cells, a process not yet fully understood, underscores the necessity for more in‐depth study. Its mechanisms remain elusive, presenting an area ripe for further exploration. Conversely, the progressive lysis of *N. gonorrhoeae* cells induced by β‐lactam exposure has been observed in *E. coli*, in which the turgor pressure resulting from the disruption of rigid bacterial cell wall was proposed as the driver to cell death.^[^ ^24^, ^29^ ^]^ These insights suggest that such pressure imbalance could be a widespread strategy employed by β‐lactam antimicrobials to initiate bacterial lysis. Although DNA fragmentation is commonly recognized as a biochemical hallmark of apoptosis in eukaryotic cells, its presence in bacteria has been viewed with skepticism. Nevertheless, mounting evidence suggests that bacterial cell death, whether induced by lethal stresses such as antimicrobial exposure or occurring naturally in processes such as autolysis, also manifests biochemical hallmarks of apoptosis including DNA fragmentation.^[^ ^23^, ^30^, ^31^, ^32^, ^33^ ^]^ Our observations in *N. gonorrhoeae* lend further support to these perspectives.

Considering the observed DNA fragmentation, we suggest that the quick onset of abrupt bacterial lysis plays a crucial role in the efficacy of our AST method shortly after antimicrobial exposure. Abrupt bacterial lysis results in the reduction of cell number in the antimicrobial‐treated group, whereas the untreated group exhibits a cell number increase due to asynchronous bacterial growth, despite the exposure duration being shorter than the typical cell replicating time. This dynamics results in a noticeable difference in total bacterial counts after one hour, with reduced counts in the treated group compared to the untreated group. Furthermore, we posit that abrupt lysis is the primary mechanism for releasing fragmented DNA into the medium, allowing for the detection of these DNA fragments within the brief antimicrobial exposure period. Although it is possible that the release of DNA fragments is attributed to changes in cell envelope permeability following antimicrobial exposure, it is noteworthy that the cell envelope of *N. gonorrhoeae* includes an outer membrane serving as a barrier to biomolecules. While β‐lactam antimicrobials can disrupt the peptidoglycan cell wall, the integrity of the outer membrane, in principle, should be minimally affected, thereby preventing the unimpeded passage of DNA fragments.^[^ ^25^, ^34^ ^]^

Agar dilution, the traditional gold standard for AST in *N. gonorrhoeae*, demands several days for results and is confined to specialized microbiology labs.^[^ ^35^ ^]^ This delay in diagnosis often leads to the premature use of broad‐spectrum antimicrobials, compromising patient care and contributing to the rise AMR. As a result, there is a growing shift from culture‐based techniques to NAATs, such as PCR, to speed up diagnosis, enabling direct AST from clinical samples without the prerequisite bacterial culture.^[^ ^36^, ^37^, ^38^ ^]^ Despite the focus on genotypic AST methods that detect resistance genes, their reliability is questionable due to the complexity of bacterial resistance mechanisms, which can render them ineffective in predicting susceptibility.^[^ ^20^, ^21^, ^22^ ^]^ Recent advances in molecular assays for phenotypic AST, utilizing NAATs to measure bacterial DNA replication as an indicator of growth, have shown promise.^[^ ^39^, ^40^, ^41^, ^42^ ^]^ However, the effectiveness of these methods in detecting susceptibility to β‐lactams in *N. gonorrhoeae* is limited by the slow replication rate of bacterium and the minimal impact of β‐lactams on DNA replication over short durations (Figure S10, Supporting Information).^[^ ^43^ ^]^ Our previous research indicated that this phenotypic approach requires extended exposure (4–6 h) to PEN^[^ ^44^ ^]^ or CEF,^[^ ^45^ ^]^ underscoring the need for innovative strategies that rely on additional biological insights for faster AST.^[^ ^34^ ^]^

Although our current study focuses on a proof‐of‐concept validation of the dual‐digital PCR approach using pre‐cultured *N. gonorrhoeae* cells, we envision that it holds potential for rapid, pre‐culture‐free AST in clinically relevant settings. Our assay, with a detection sensitivity of 4 × 10^3^ CFU mL^−1^ (Figure S11, Supporting Information), is sufficient for a substantial portion of sample types, such as urine, vaginal swabs, anorectal swabs, and penile swabs, which typically have bacterial loads between 10^4^ and 10^5^ CFU mL^−1^.^[^ ^16^, ^46^, ^47^ ^]^ In these cases, swab samples could be directly eluted into a bacterial culture medium for antimicrobial exposure, followed by dual‐digital PCR using cell suspension. For urine samples, a centrifugation‐assisted buffer exchange into a cell culture medium could be employed. In cases where the bacterial load in the swab or urine is below our detection limit, we can incorporate a sample processing step like centrifugation for bacterial enrichment. Additionally, the detection sensitivity of our array could be enhanced by increasing its analytical volume, facilitating the detection of more bacteria in a sample. Moreover, while our current microfluidic array is limited to small‐batch fabrication, it could potentially be adapted for mass‐manufacturing by using micro‐injection molded high optical clarity cyclo‐olefin polymer and sealing it with a semi‐gas‐permeable thermoplastic thin film, enhancing its suitability for clinical applications.^[^ ^48^ ^]^ Of note, although OD measurement is useful in differentiating susceptible and resistant *N. gonorrhoeae* after antimicrobial exposure, it is not suitable for clinical implementations. First, this method is incapable of identifying unknown pathogens in a sample. Second, it requires a high initial bacteria concentration for generating detectable signals, often beyond clinically relevant levels. Moreover, in clinical samples containing commensal bacteria or multiple pathogens, OD measurement captures the growth of all bacterial species present, therefore lacking the specificity for assessing the antimicrobial susceptibility of the target pathogen.

Our approach, initially developed for *N. gonorrhoeae* AST against β‐lactam antimicrobials, has the potential for wider application across various bacterial species and antimicrobial classes. Studies have shown that other antimicrobials, such as fluoroquinolones and aminoglycosides, can also induce DNA fragmentation in bacteria, suggesting the broad relevance of our findings.^[^ ^23^, ^31^ ^]^ The role of abrupt lysis in releasing fragmented DNA, while promising, requires further investigation across different bacteria and antimicrobials.^[^ ^49^ ^]^ Nevertheless, techniques to increase cell envelope permeability, such as chemical or physical methods,^[^ ^50^, ^51^, ^52^ ^]^ could potentially aid in releasing fragmented DNA, though maintaining cell integrity is crucial. These possible applications are especially pertinent for the ESKAPE pathogens,^[^ ^53^ ^]^ which are identified as critical multidrug‐resistant bacteria in urgent need of rapid AST and effective therapies. As a valuable next‐step, we propose assessing our dual‐digital PCR approach for carbapenem‐resistant *Enterobacteriaceae* (CRE) infections. CRE represents a global health threat that urgently demands rapid AST solutions.^[^ ^54^ ^]^ Given that carbapenems are strong beta‐lactam antibiotics and *Enterobacteriaceae*, such as *E.coli* and *Klebsiella pneumoniae*, are Gram‐negative bacteria, similar bacterial responses might be observed. Moreover, in implementing our AST approach, employing multi‐copy genes as PCR targets is advantageous in establishing a clearer distinction in DNA fragment copies between antimicrobial‐treated and ‐untreated groups, thereby enhancing the precision and reliability of our assay. Additionally, our approach is particularly beneficial for AST in bacterial species not prone to autolysis, which results in a reduced background of DNA fragments. Ultimately, our study aims to advance the development of new NAAT‐based strategies for rapid phenotypic molecular AST, contributing valuable insights in the fight against AMR.

## 4 Experimental Section

### Bacteria Strains

Reference stains ATCC 43069 and ATCC 49226 were purchased from the ATCC(VA, USA). Clinical isolates 16‐03‐16, 16‐05‐05, 16‐04‐03, 16‐10‐15, 16‐03‐15, and 16‐04‐11 were collected in Baltimore, MD as part of routine surveillance projects in 2016.^[^ ^55^ ^]^ Four WHO reference strains including WHO‐G, WHO‐L, WHO‐Z, and WHO‐X^[^ ^56^ ^]^ were obtained from the CDC & FDA Antibiotic Resistance Isolate Bank (https://wwwn.cdc.gov/ARIsolateBank/Panel/PanelDetail?ID=11). All the *N. gonorrhoeae* strains used in this study are listed in Table S1 (Supporting Information).

### Reagents

Antimicrobials were purchased from Acros Organics (Belgium). The Apo‐Direct kit was purchased from BD Bioscience (USA). All components for GW medium including medium 199, dextrose, ammonium bicarbonate, sodium acetate trihydrate, *l‐*glutamine, spermidine, *l*‐arginine, hypoxanthine, uracil, oxaloacetate, thiamine hydrochloride, *l*‐ornithine, nicotinamide adenine dinucleotide, and sodium *d, l*‐lactate were purchased from MilliporeSigma (St. Louis, MO, USA). The medium was then prepared according to the formula described by Wade and Graver.^[^ ^57^ ^]^ *E*‐test strips were purchased from BioMérieux (Marcy‐I’Étoile, France). TaqMan Gene Expression Master Mix (2X), Amplitaq Gold DNA polymerase (Ultrapure), Tween‐20, and Nucleic‐free water were purchased from Thermo Fisher Scientific (Waltham, MA, USA). Bovine Serum Albumin (BSA, 20 mg mL^−1^) was purchased from New England Biolabs Inc (Ipswich, MA, USA). Primers and probes for *opa* and *terminus* genes were synthesized by Integrated DNA Technologies Inc. (Coralville, IA, USA) and dissolved in Nuclease‐free water to a final concentration as 100 µm for long‐term storage. Sequences of primers and probes used in this study were summarized in Table S2 (Supporting Information).

### Bacteria Culture, Growth, and Minimum Inhibitory Concentration (MIC) Determination

Each *N. gonorrhoeae* strain was resuspended in GW medium containing 20% glycerol (v/v) and frozen at −80 °C for long‐term storage. At the time of use, isolates were streaked from glycerol stocks onto Chocolate II Agar plates (BBL, Becton Dickinson) and grown overnight in a 35 °C incubator with 5% CO_2_. Single gonococcal colonies were then sub‐cultured at least once onto fresh Chocolate II Agar plates and incubated at the same condition as mentioned above before use. Next, several colonies were scraped from plates and resuspended in 1 mL pre‐warmed (35 °C) GW medium. Bacteria concentration was determined via optical density measurement at 600 nm on a spectrometer, and the cell samples were diluted to create a 2‐mL working suspension matching to 5 × 10^5 ^CFU mL^−1^ in 50‐mL conical tubes. For the antimicrobial susceptibility testing of each strain, two 2‐mL working suspensions were prepared, one was treated with antimicrobials, and the other one served as a no drug control. The working suspensions were then agitated at 180 rpm on an orbital shaker within the incubator. Samples were collected after cultivation for downstream analysis.

The MICs of each *N. gonorrhoeae* strain were determined to the antimicrobials used in this study via E test. Bacteria were first resuspended in GW medium, and the turbidity was adjusted to 0.5 McFarland standard. Bacteria were then inoculated onto Agar plates using sterile swabs, and the E test strips were positioned upright on the center of the plates. After overnight incubation, the MIC values of each strain were read at the scale where the pointed end of the eclipse intersected the strips, indicating the antimicrobial concentration that completely inhibited cell growth. The MIC values of each strain to antimicrobials are listed in Table S1 (Supporting Information).

### Device Fabrication

The multi‐layer PDMS device was fabricated as previously described with slight modifications.^[^ ^27^ ^]^ Briefly, two reusable master molds, one with a microarray pattern and the other one with a pattern for the suction layer were prepared using the standard photolithography technique on two 4‐inch silicon wafers. Then the SYLGARD 184 Silicone Elastomer Kit (Dow Corning, Midland, MI) was used to fabricate the PDMS components. Specifically, this PDMS device was assembled by four layers including the suction layer, a 100‐µm‐thick PDMS membrane, the microarray layer, and a glass coverslip. Each chip consisted of two identical modules, and each module features an inlet with multi‐level bifurcated microchannels connecting to 2048 dead‐end microwells. Each microwell was 200 µm in length, 110 µm in width, and 300 µm in height, making each module capable of analyzing up to 12 µL of samples. Detailed procedures for device fabrication are described in the Supporting Information.

### Real‐Time OD_600_ Measurement on Plate Reader

For each strain, several gonococcal colonies were scraped from agar plates, resuspended in GW medium, and adjusted to OD_600_ as 0.1. On a 96‐well plate, 200 µL bacteria suspensions were added into each well, followed by the addition of antimicrobial (PEN or CEF) with different concentrations except the no drug control. The plate was then positioned on the plate reader, where bacteria suspensions were grown at 35 °C with continuous orbital shaking. OD_600_ values of each well were recorded every 10 min.

### Dual‐Digital PCR

Dual‐digital PCR was performed on the microfluidic array with multi‐step sample loading capability. To minimize PCR buffer‐induced *N. gonorrhoeae* lysis, bacteria and PCR reaction buffer were loaded into the chip at two separate stages. An 8‐µL PCR reaction mixture was composed of 5 µL TaqMan Gene Expression Master Mix (2×), 0.45 µL *opa* forward primer (20 µm), 0.45 µL *opa* reverse primer (20 µm), 0.45 µL *terminus* forward primer (20 µm), 0.45 µL *terminus* reverse primer (20 µm), 0.125 µL *opa* probe (20 µm), 0.125 µL *terminus* probe (20 µm), 0.5 µL BSA (20 mg mL^−1^), 0.25 µL Amplitaq Gold DNA polymerase (5 U µL^−1^), 0.1 µL tween‐20 (10%), and 0.1 µL nuclease‐free water.

After bacteria cultivation in the 2‐mL working suspensions, the bacteria suspension was diluted fivefold using fresh GW medium, and 2.5 µL of the diluted bacteria sample was digitalized into the chip to maximize the occurrence of a single‐target event in individual partitions. Next, an 8 µL PCR mixture was loaded into the chip, immediately followed by oil partitioning to form discrete microwell units for PCR reactions. For experiments testing the cell culture supernatants, 500 µL of bacteria suspension was centrifuged at 1000 × *g* for 15 min. The supernatant was then diluted for fivefold and 2.5 µL of the diluted supernatant was loaded into the chip for analysis as described above. The PCR thermocycling program was as follows: 50 °C for 2 min, 95 °C for 10 min, 10 s at 95 °C and 60 s at 60 °C for 60 cycles.

### Bacterial Detection Limit on Microfluidic Array

To evaluate the detection sensitivity of this approach, fivefold serially diluted *N. gonorrhoeae* culture cells (Strain: ATCC 43069) were tested in GW medium at concentrations of 10^5^, 2 × 10^4^, 4 × 10^3^, 8 × 10^2^, and 0 CFU mL^−1^, corresponding to 250, 50, 10, 2, and 0 CFU as 2.5 µL of cell sample was loaded into this array. Then the dual‐digital PCR targeting *opa* and *terminus* genes were performed. Microwells showing positive signals for both genes indicated intact *N. gonorrhoeae* cells, which were counted for bacterial quantification. The detection limit was established at the concentration that reliably detected *N. gonorrhoeae* cells without significant impact of Poisson noise.

### Chip Imaging and Image Processing

Full‐view fluorescent chip images were captured using the in‐house built four‐color imaging platform (FAM, HEX, Texas Red, and Cy5). The setup was composed of a commercial flatbed heater (Bulldog Bio) for thermal control, LED sources (Quadica Development Inc.) with corresponding excitation filters (Omega Optical) for illumination, and a 12 M pixel CMOS Mirrorless camera (Sony) with a 50‐mm macro focusing lens (Sony) attached to a filter wheel (QHYCCD), which was mounted with emission filters (Omega Optical), for capturing fluorescence images. The imaging platform was controlled with an Arduino microcontroller (Arduino), which was interfaced by a Java‐based Graphical User Interface (Figure S12, Supporting Information). Images were then analyzed using a customized MATLAB pipeline (Figure S13, Supporting Information). Detailed procedure for image processing is described in Supporting Information.

### Live‐Cell Microscopy

Microscopy experiments were performed using a Zeiss LSM 780 inverted laser scanning confocal microscope (Zeiss, Jena, Germany) outfitted with a full incubation chamber for regulating temperature and CO_2_ to enable live cell imaging. A microliter‐sized fluid chamber (Figure S14, Supporting Information) was devised on a glass coverslip to immobilize bacteria and prevent evaporation during imaging. Specifically, cells were resuspended in GW medium and adjusted to a concentration as 5 × 10^8 ^CFU mL^−1^. Then 2 µL bacteria suspension was pipetted onto a glass coverslip (No. 1, 35 mm × 50 mm), and a small piece of 1 mm‐thick agarose pad (1%) made from Mueller Hinton II (MH‐II) broth was placed on top of the cells. Next, a 1 mm‐thick hollow square PDMS slab was placed around the agarose pad as a fence, and the interior space was filled with 200 µL GW medium. Finally, a glass coverslip (No. 1, 20 mm × 20 mm) was attached on top to seal the fluid chamber. Cells were imaged immediately afterward so that the dwelling time from sample preparation to cell imaging was ≈5 min. On the microscope, cells were imaged using Plan‐Apochromat 63×/1.4 oil DIC (Differential Interference Contrast) objective (Zeiss, Jena, Germany) with enhanced contrast under brightfield. Cell images were captured every 5 min on Zeiss ZEN Black software with 1.5× optical zoom. Fiji was used for processing the images and then converted the sets of images to videos via images stacking. The videos were also stabilized in iMovie to correct the microscope drift as necessary.

### TUNEL Assay

Labeling of DNA fragments in *N. gonorrhoeae* cells was performed using Apo‐Direct Kit, which employs FITC‐conjugated deoxy uridine triphosphate (FITC‐dUTP) for the staining of 3′‐hydroxyl ends of DNA breaks and uses PI as a counterstain. For detection, cells were analyzed on a BD FACSCanto Flow Cytometer. In this experiment, 488 nm excitation source, 530/30, and 585/42 nm emission filters were used. PMT voltage settings used were as follows: 450 (FSC), 700 (SSC), 350 (FITC), and 400 (PI). For each sample, 100000 events were recorded, and data were analyzed using FlowJo software. See the Supporting Information for details of cell treatment, staining, and data analysis.

### Statistical Analysis

All statistical data presented in this work were analyzed using GraphPad Prism (GraphPad Software Inc., San Diego, CA), with error bars on each datapoint or bar representing the mean and SD. The *p*‐value reported in Figure 1A was calculated by performing an unpaired two‐tailed *t*‐test. GraphPad Prism was also used to fit the linear regression curves and to extract relevant fit parameters in Figure S5 (Supporting Information).

## Conflict of Interest

The authors declare no conflict of interest.

## Supporting information



Supporting Information

Supplemental Video 1

Supplemental Video 2

Supplemental Video 3

## Data Availability

The data that support the findings of this study are available in the supplementary material of this article.
