# Supplementary material for: Exploiting β‐Lactams‐Induced Lysis and DNA Fragmentation for Rapid Molecular Antimicrobial Susceptibility Testing of Neisseria Gonorrhoeae via Dual‐Digital PCR
Source: Adv Sci (Weinh). 2024 Oct 18;11(46):2405272. doi: 10.1002/advs.202405272 (PMC11633544; doi:10.1002/advs.202405272)
Supplement: Supplementary file 1 — Supporting Information [file ADVS-11-2405272-s001.docx]

**Supporting information**

**Exploiting β-lactams-induced lysis and DNA fragmentation for rapid molecular antimicrobial susceptibility testing of *Neisseria gonorrhoeae* via dual-digital PCR**

Jiumei Hu^1^, Liben Chen^1^, Pengfei Zhang^2^, Fan-En Chen^2^, Hui Li^1^, Kuangwen Hsieh^1^, Sixuan Li^1^, Johan H. Melendez^3^ and Tza-Huei Wang^1, 2, 4, *^

^1^Department of Mechanical Engineering, Johns Hopkins University, Baltimore, MD 21218, USA

^2^Department of Biomedical Engineering, Johns Hopkins School of Medicine, Baltimore, MD 21205, USA

^3^Division of Infectious Diseases, Department of Medicine, Johns Hopkins University School of Medicine, Baltimore, MD 21205, USA

^4^Institute for NanoBiotechnology, Johns Hopkins University, Baltimore, MD 21218, USA

*Corresponding author: Email: thwang@jhu.edu, Tel: (+1) 410-516-7086

**Supplementary Notes**

***Device fabrication***

Two reusable master molds for replicating PDMS microarray layer and suction layer were fabricated by standard photolithography technique on 4-inch silicon wafers (Polishing Corporation of America, Santa Clara, CA). Photomasks were designed using AutoCAD software and printed by CAD/Art Services, Inc. (Bandon, OR, USA). For the microarray mold fabrication, we designed different heights for microchannels (100 µm) and microwells (300 µm). First, a 100-µm-thick SU8-3050 photoresist (Microchem Corp., Newton, MA) was spun-coated onto a dehydrated wafer at 1500 rpm for 35 s. After 30 min of soft bake, the wafer was attached onto the photomask with microchannel pattern and exposed under UV light on the aligner, followed by post exposure bake at 95 °C for 3 min. Next, another two layers of 100-µm-thick SU8-3050 photoresist were spun-coated onto silicon wafer. After 1 h of soft bake, the wafer was attached onto the photomask with microwell pattern, and then exposed under UV light after alignment. The wafer was post-exposure baked at 95 °C for 15 min, and then developed in SU-8 developer (MicroChem Corp., Newton, MA) for 20 min to generate the solid pattern of microarray. For the mold of suction layer, a single-layer SU8-3050 was applied onto a dehydrated silicon wafer to achieve a thickness of ~100 µm. Photolithography steps including soft bake, UV exposure, post exposure bake, and development were then conducted to generate the pattern on the wafer. Finally, the two molds were hard baked at 200 °C for 1h.

Molds were treated with chlorotrimethylsilane (Sigma Aldrich, St. Louis, MO) for 10 min to facilitate smooth demolding of PDMS before use. First, 10:1 (w/w) PDMS (SYLGARD 184 Silicone Elastomer Kit, Dow Corning, Midland, MI, USA) was spun onto the microarray mold at 100 rpm for 2 min, followed by 5-min degasification to remove air bubbles and 15-min baking in an 80 °C oven to cure the PDMS. To make the thin PDMS membrane, 5 g 5:1 (w/w) PDMS was spun-coated onto a blank wafer at 700 rpm for 35 s (~100 µm in thickness) and baked at 80 °C for 6 min. To peel off the PDMS membrane, we put another thicker blank PDMS onto the wafer as a sacrifice layer, baked at 80 °C for 6 min and then strip off the stacked PDMS. The reusable PDMS suction layer was fabricated by pouring ~30 g 10:1 (w/w) PDMS onto the mold, followed by air bubble removal in a vacuum chamber and baking at 80 °C for 40 min. All the PDMS components were cut into regular shapes and punched with holes to access fluidic inlets. The sandwiched microarray was then plasma-bonded with glass coverslip and the thin PDMS membrane. Two punched PDMS adaptors were also bonded onto the inlet of the thin membrane to fit the pipettor tips during sample loading. The assembled device was baked at 80 °C overnight. Suction layer was applied onto the device each time before use.

***Image processing***

Image processing was performed using customized MATLAB code. The images were first read, cropped, and then de-skewed via a projection-based skew correction. During skew correction, the image was preprocessed via top-hat filtering to eliminate uneven illumination and median filtering to remove background noise, which was then converted to a binary image. On the binarized image, a horizontal projection profile was generated, which is a vector with the number of elements equals to the rows of the image and each elements represents the sum of pixels in each row. When an image is tilted, the values of most elements are non-zero. We then rotated the image over a range of angles (e.g. -10° to 10°) with a unit step as 0.01° until the maximum number of elements with a value of zero was reached. Next, a similar projection-based gridding method was adopted for mask generation to realize the positioning of each microwell. Specifically, we first did horizontal projection on the de-skewed image (binarized), which returned a vector indicating the sum of pixels in each row. Using the inbuilt “findpeaks” function in MATLAB, rows with local maximum pixel intensities larger than their two neighboring rows were recorded, along with the indices where the local maxima occur. We then manually set up a threshold, which defined the distance between each local maxima, to filter out non-target peaks and therefore get the x-coordinates of each microwell. Next, a same workflow with vertical projection was performed to get the y-coordinate of each microwell so that a mask was generated for downstream analysis. After extracting the pixel intensities, each microwell was indexed by its position (row, column), and positive signals were differentiated from background. In our dual-digital PCR assay, a FAM channel image (target: *opa* gene) and a cy5 channel image (target: *terminus* gene) were processed separately as described above, which generated two matrices with same dimensions showing the location of positive microwells. We then converted the matrices into logical matrices with entries from the Boolean domain B = {0, 1}, where the positive microwells were denoted as “1”, and negative microwells were denoted as “0”. By doing logical matrices computation, microwells that detected only *opa* gene, *terminus* gene, or both genes could be distinguished, and different kinds of microwells were colored in green, red, and yellow (***Figure S13***).

***TUNEL assay***

First, several *N. gonorrhoeae* colonies (ATCC 43069) were scraped from agar plate, resuspended in 5 mL MH-II medium to 10^6 CFU/mL and cultured overnight with continuous shaking at 180 rpm. In the next day, cells were inoculated into fresh medium to 10^7 CFU/mL and sub-cultured for another 4 hours before use. Cell density was then measured via spectrometer and the concentration was adjusted to 10^7 CFU/ml for downstream experiments. In two 50-mL conical tubes, one sample was exposed to 0.2 µg/mL PEN and the other one served as no drug control. After 1 h drug treatment, 1 mL cell suspension was collected from each tube and cells were spun down at 6500 xg for 5 min in a refrigerated centrifuge. Cells were then washed twice with 1x ice-cold, filtered PBS (pH 7.4). During the cell fixation, 1 mL 4% Paraformaldehyde in PBS (pH 7.4) was added to resuspend the cell pellet, which was then incubated at room temperature for 30 min. Cells were spun down at 6500 xg for 5 min to remove the supernatant, and 500 μL ice-cold permeabilization solution (0.1% Triton X-100 and 0.1% sodium citrate in PBS) was added into each tube, which were placed on ice for 2 min. 1 mL ice-cold 1X PBS was then added into each tube immediately to terminate the permeabilization. Cells were spun down at 6500 xg for 5 min and washed once in 1 mL wash buffer (kit component). For FITC staining, 50 µL DNA labeling solution (composed of Reaction buffer, deoxynucleotidyl transferase enzyme, FITC-dUTP, and distilled water) was added to resuspend the cell pellet, and the tubes were incubated at 37 °C in the dark for 1.5 h. At the end of incubation, 1 mL rinse buffer (kit component) was added into each tube to terminate the staining reaction. Cells were spun down at 6500 xg for 5 min and then rinsed again using 1 mL rinse buffer. Finally, 500 µL PI/RNase staining buffer was added into each tube, which were wrapped in foil and incubated at room temperature for 30 min before running the flow cytometry. The PI/RNase solution can reduce RNA-related signal, and non-DNA-containing particles can be electronically gated away from cells (DNA-containing population) to minimize unwanted background. For the unstained sample or sample stained only with PI, the FITC staining step was omitted and cells were resuspended in 500 µL PBS or 500 µL PI/RNase staining buffer in the end.

Cell samples were analyzed on BD FACSCanto Flow Cytometer equipped with ﻿FACSDiva software. The FACSCanto is built with blue (488 nm) and red (633 nm) lasers for excitation and different emission filters that enable the acquisition of up to six fluorescent signals as well as two scatter parameters (FSC and SSC). In our case, 488 nm excitation source, 530/30 nm (for FITC fluorescence) and 585/42 nm (for PI fluorescence) emission filters were used. Data were collected at low flow rate, and the following PMT voltage settings were used: 450 (FSC), 700 (SSC), 350 (FITC), and 400 (PI). Beads calibration was performed every time before the experiment. For each sample, 100,000 events were recorded, and data were analyzed using FlowJo software. Single cells were first gated via the “Area Scaling” scheme by plotting the area of FSC pulse (FSC-A) against the height (FSC-H), where all singlets were clustered diagonally and separated from cell doublets or clumps. Fluorescence intensities of FITC and PI in unstained cell sample were set to be the background. Cells displaying FITC and PI fluorescence intensities above background (FITC+, PI+) were identified as TUNEL-positive signals.

Stained cell samples were also analyzed *in situ* on Zeiss LSM780 confocal microscope. Cell suspensions were spun down at 6500 xg for 5 min to remove supernatant and pellets were resuspended in 10 µL PBS. 2 µL of the concentrated cells were sandwiched between a glass coverslip (No. 1, 35 mm x 50 mm) and a small piece of 1 mm-thick agarose pad (1%). Another glass coverslip (No. 1.5, 18 mm x 18 mm) was placed on top of the agarose pad for preventing sample evaporation. On the Zeiss ZEN Black software, cells were imaged and captured at two different channels for FITC and PI simultaneously. We then used Fiji for image processing.


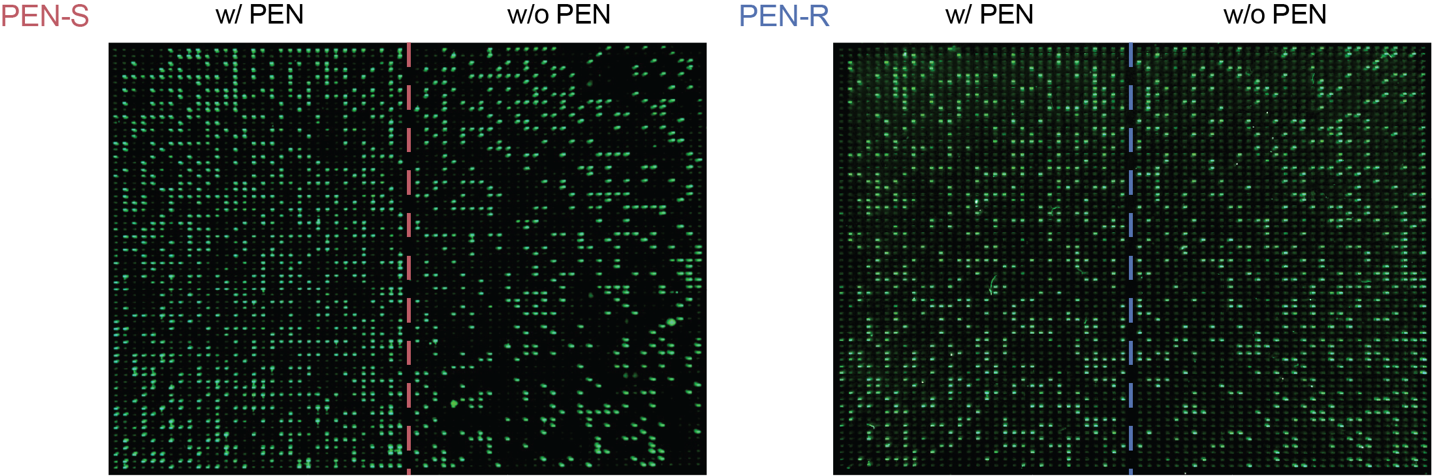


***Figure S1***. Exemplary fluorescent chip images derived from single-cell digital PCR of *N. gonorrhoeae* targeting the *opa* gene following a one-hour exposure to 2 μg/mL PEN. In the PEN-susceptible strain, the PEN-treated group exhibited a notably higher number of *opa*-positive microwells compared to the untreated group. Conversely, the treated and untreated groups of the PEN-resistant strain displayed a similar number of positive microwells. Approximately 500 cells were digitalized into the microwell array for both samples.


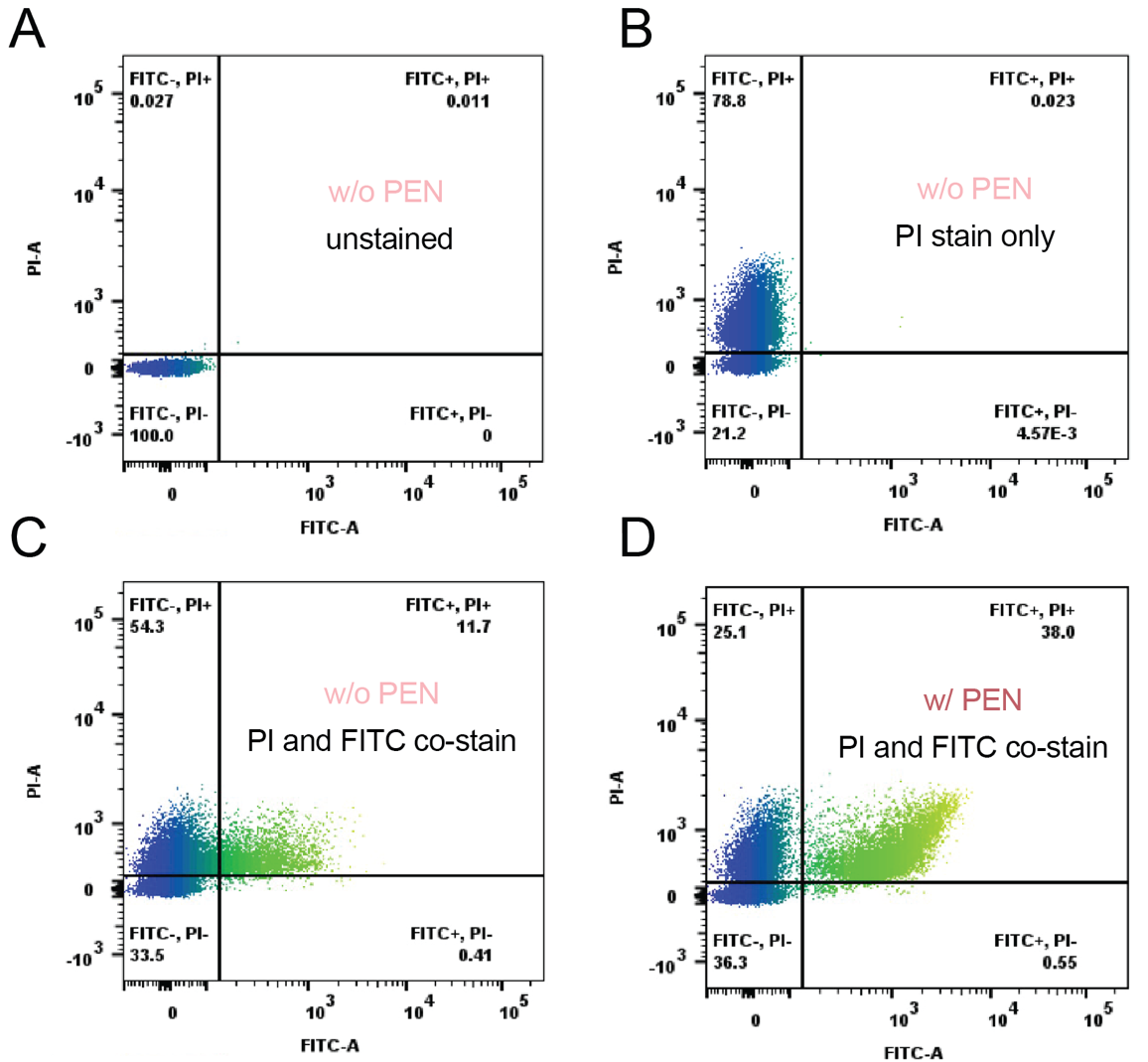


***Figure S2***. *N. gonorrhoeae* DNA fragmentation analysis on flow cytometry. (A) Untreated ATCC 43069 cells were used as an unstained control to determine the background fluorescence intensities of FITC and PI. (B) Untreated cells stained with PI only was used to evaluate the staining efficiency of PI. (C) The TUNEL-positive cells were indicated as FITC+ and PI+. Sample without PEN treatment had a TUNEL-positive percentage of 11.7%. (D) The TUNEL-positive percentage of PEN-treated sample was 38%, which is significantly higher than the untreated sample.

***
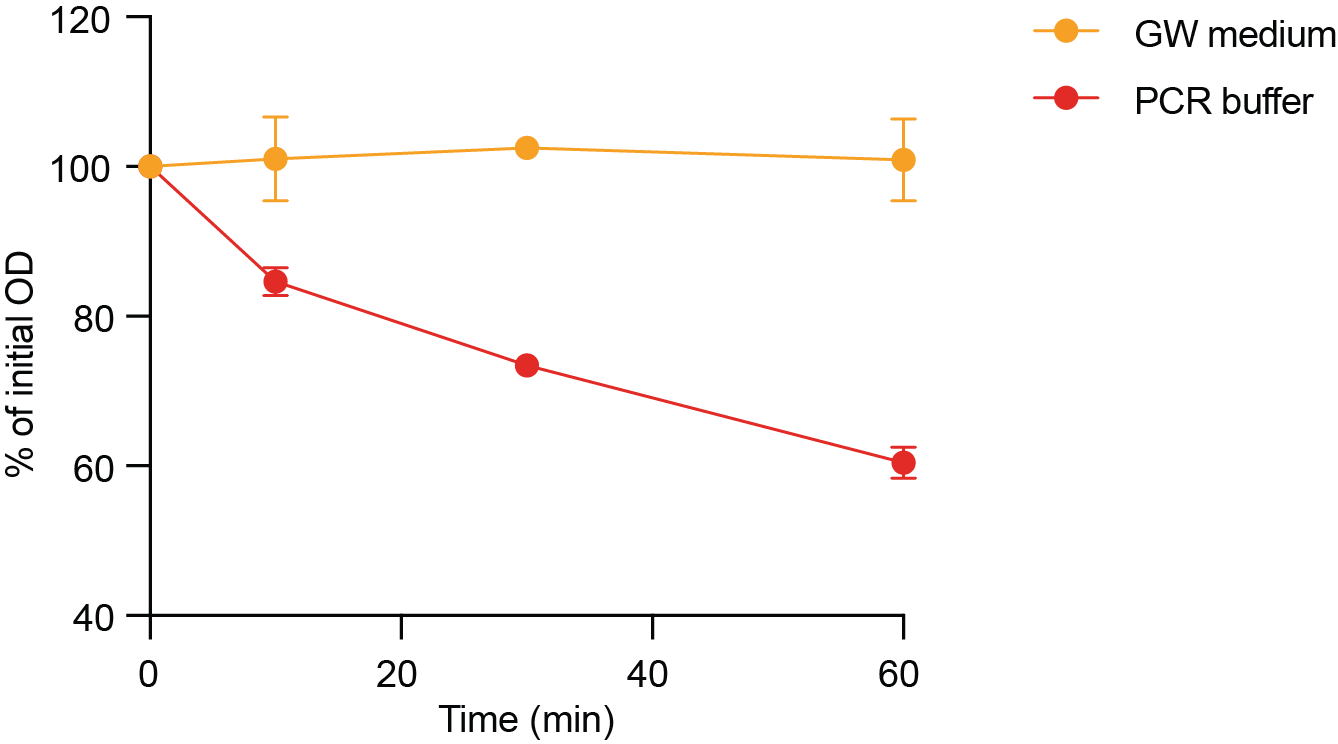
***

***Figure S3***. *N. gonorrhoeae* cells underwent lysis when mixed with alkaline PCR buffer, as indicated by the decrease of optical density overtime (n=3).


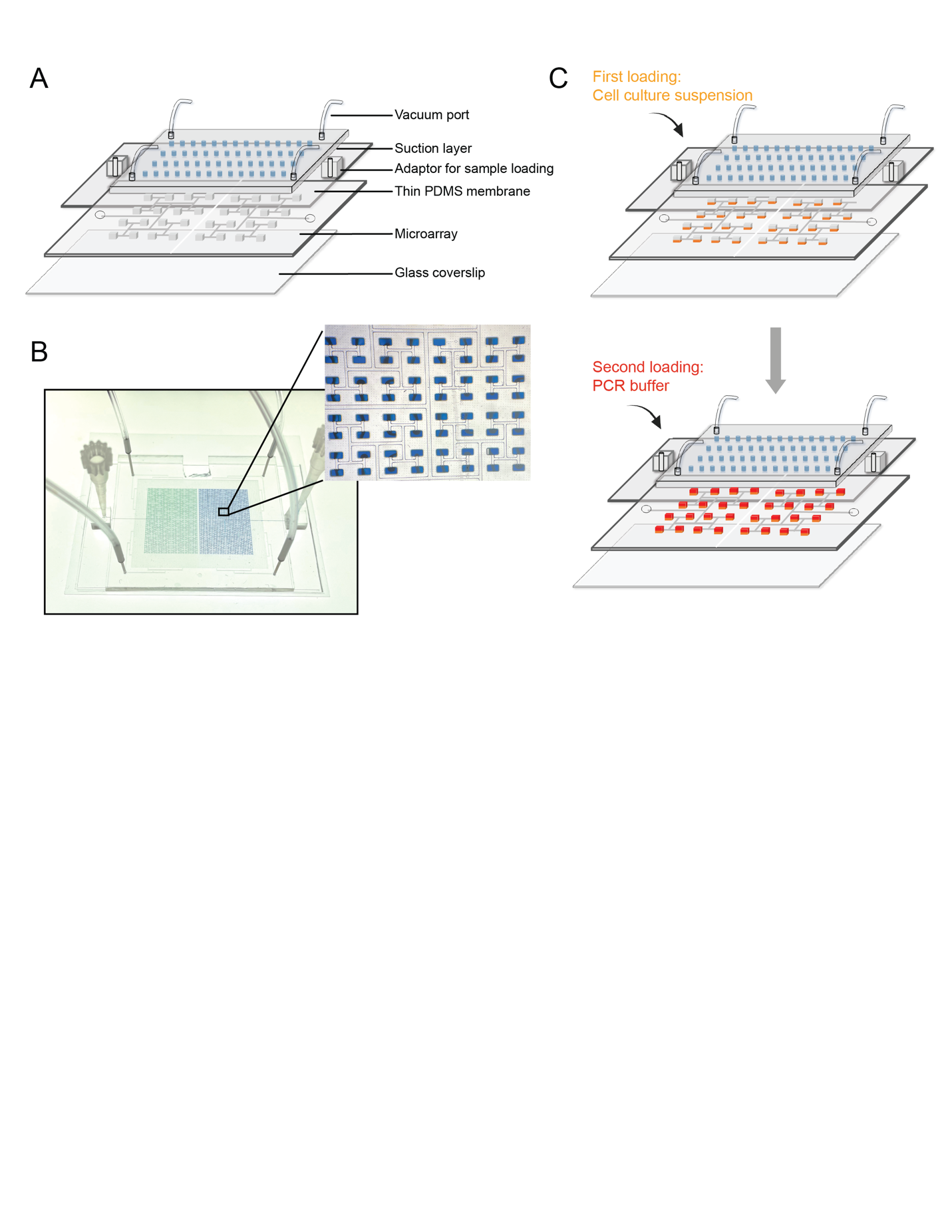


***Figure S4***. Design and operation of the multi-step loading chip. (A) The device is fabricated by assembling a suction layer, a thin PDMS membrane, a microarray layer, and a glass coverslip. The suction layer features a large cavity with a micropillar array to prevent the cavity from collapsing when connecting to external vacuum source. The microarray is composed of two identical modules. Each module contains 11-level bifurcated channels and 2048 dead-end microchambers. (B) The real image of our chip. Enlarged portion shows the bifurcated channels and dead-end microchambers. (C) Dual-digital PCR assay was performed via a two-step loading to prevent bacterial lysis induced by PCR buffer.


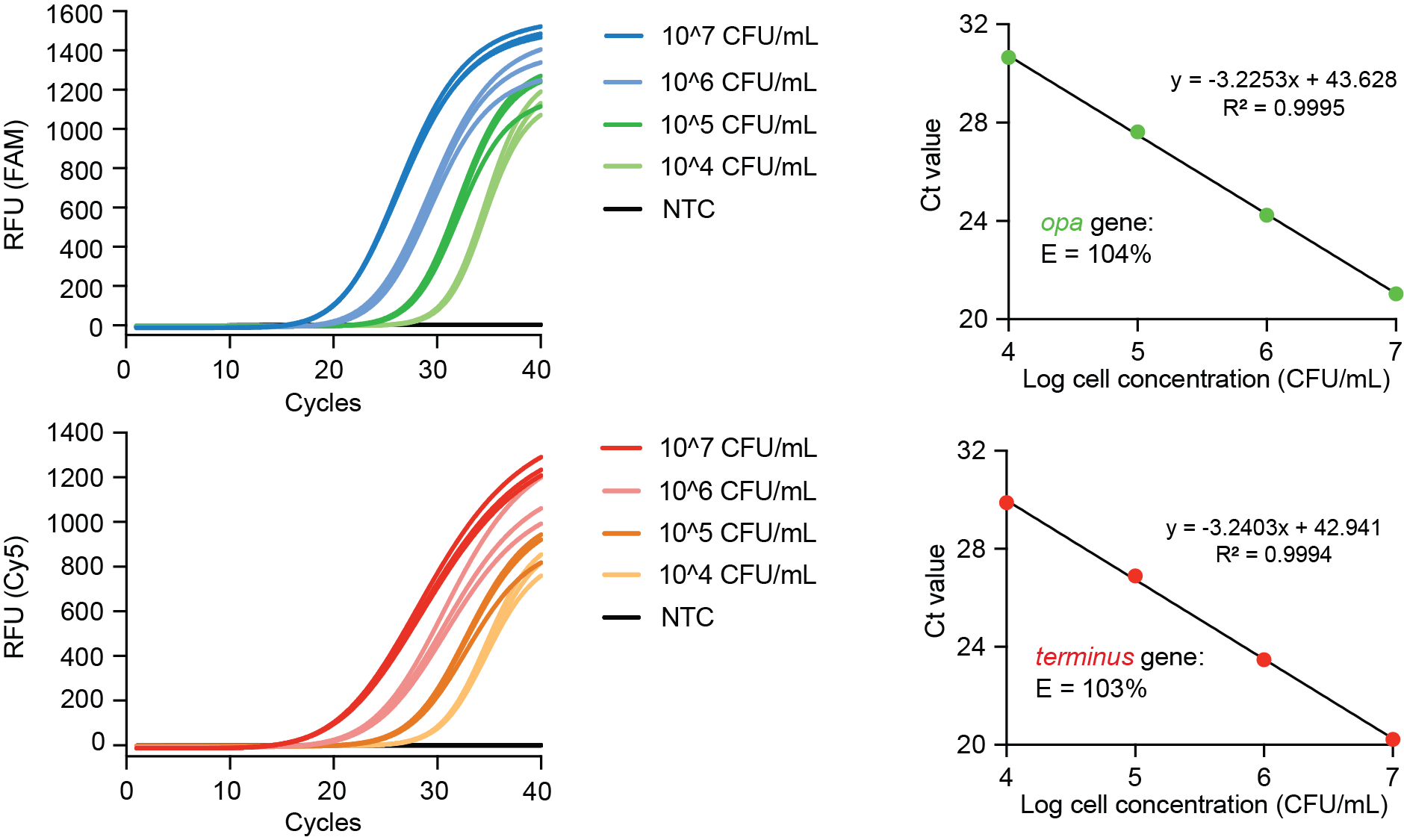


***Figure S5***. Amplification efficiency evaluation of the two-color TaqMan PCR assay was performed by preparing a dilution series of *N. gonorrhoeae* cell samples in GW medium from 10^7 CFU/mL to 10^4 CFU/mL. 1 μL of each titration was mixed with 9 μL PCR reaction buffer (contains the primers and probes for *opa* and *terminus* genes) and then directly amplified on Bio-Rad CFX real-time PCR machine. The Ct values of *opa* and *terminus* genes at each titration (n=3) were read at FAM and cy5 channel separately and were used to construct standard curves. Amplification efficiency for each target was calculated by the following equation: E=10^(-1/S)-1 (S=slope of standard curve). Both the *opa* (top) and *terminus* genes (bottom) showed good amplification efficiencies in our assay.


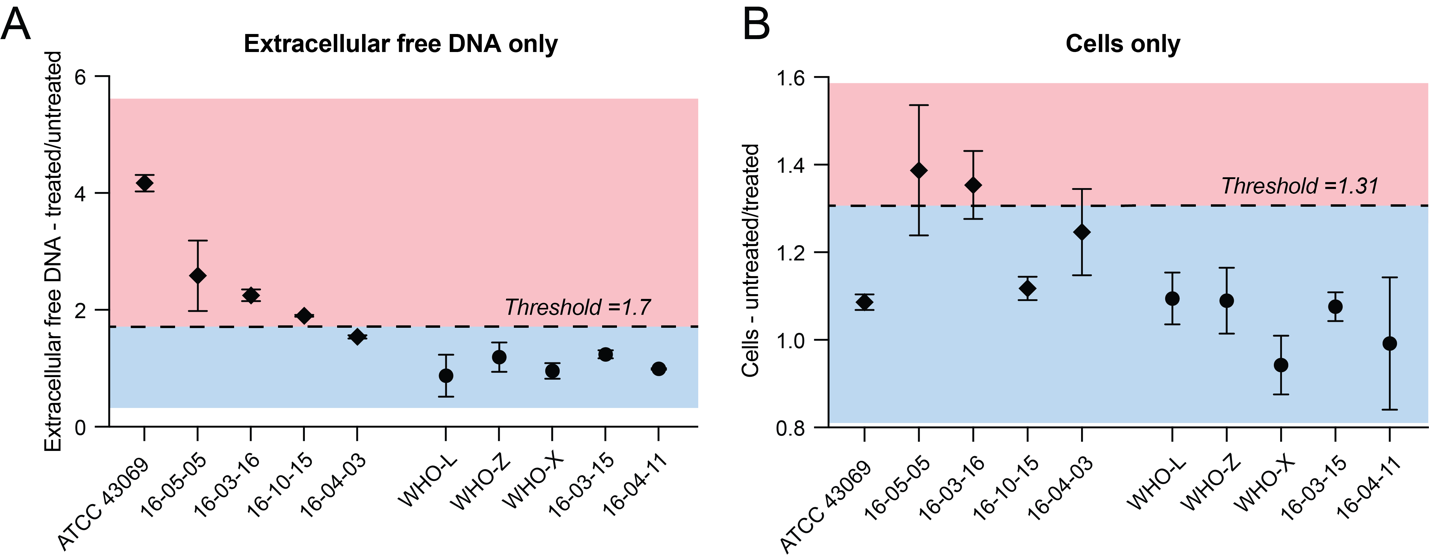


***Figure S6***. Using extracellular free DNA fragments (A) or cell number (B) as the sole metric compromised the AST performance. Each data set contains at least two replicates.


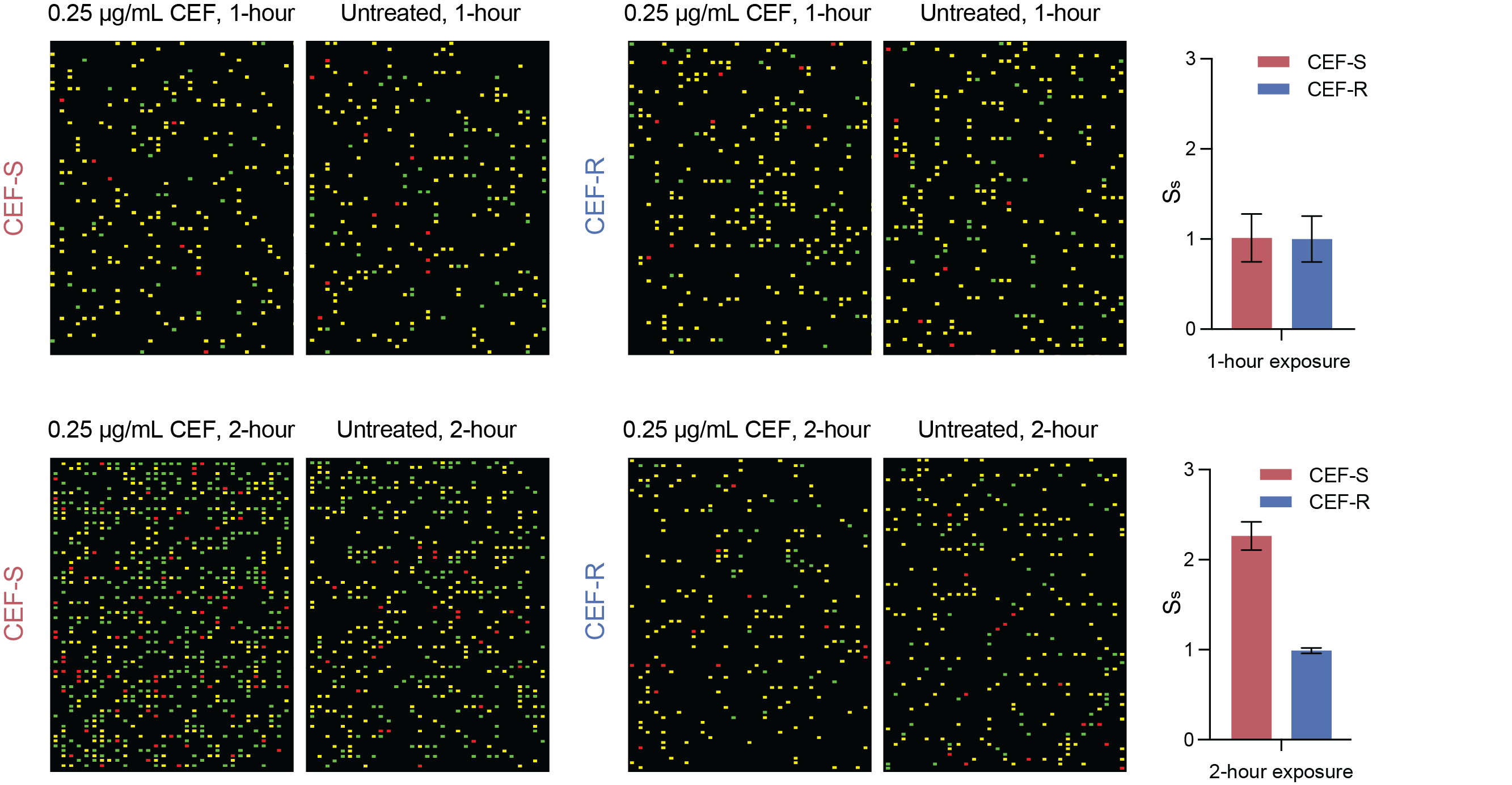


***Figure S7****.* Representative dual-digital PCR results obtained from ATCC 43069 (susceptible) and WHO-X (resistant) strains under CEF exposure at 0.25 µg/mL. With one hour exposure, the susceptible strain and resistant strains cannot be differentiated using our AST approach. However, extending the antibiotic exposure time to two hours exhibited clear difference in the susceptibility score for the two strains. Error bars: n=3.


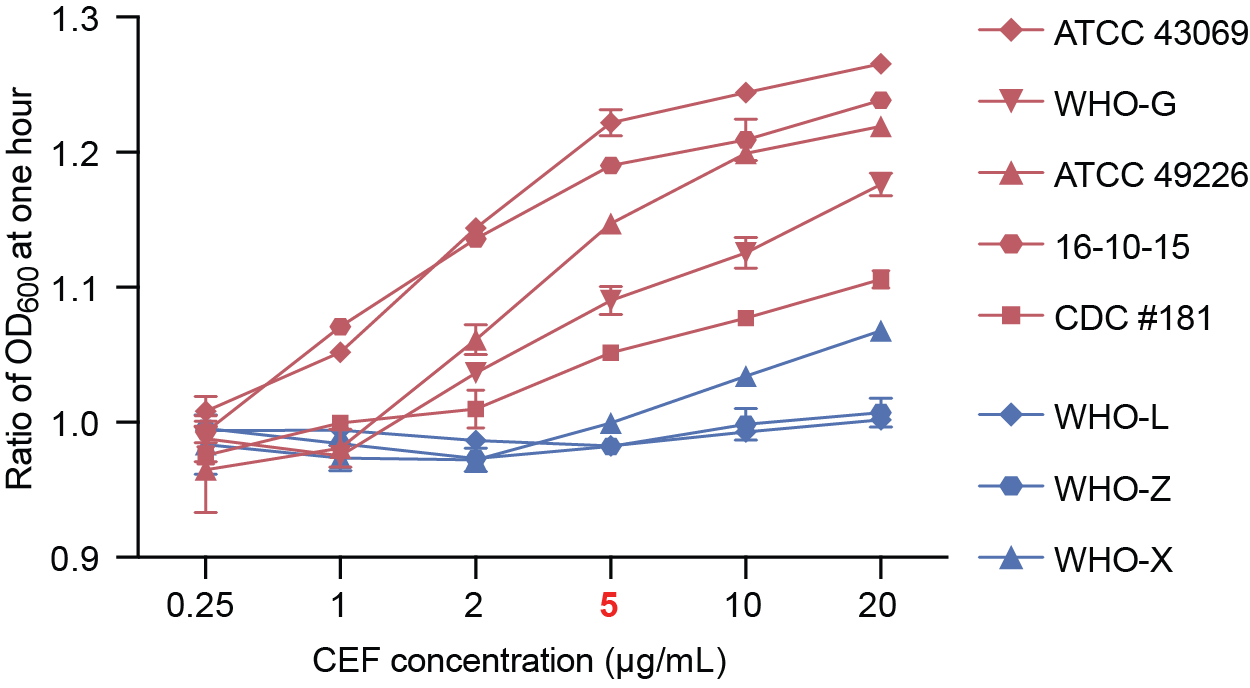


***Figure S8****.* OD measurement was employed on a panel of CEF-susceptible and -resistant *N. gonorrhoeae* strains at varying CEF titrations to identify the optimal concentration for use in our AST approach. Specifically, each bacterial strain was exposed to different CEF concentrations ranging from 0.25 µg/mL to 20 µg/mL for an hour, alongside an untreated control. The corresponding OD values of each condition were recorded. Subsequently, we calculated the OD_600_ ratio between the value obtained from untreated sample and those obtained from each CEF-treated sample independently, denoting as “Ratio of OD_600_ at one hour” on y axis. These ratios were then utilized to evaluate the impact of different CEF concentrations on bacterial growth, where a ratio exceeding one indicates the cell growth inhibition resulting from antimicrobial treatment. Each datapoint contains three replicates.


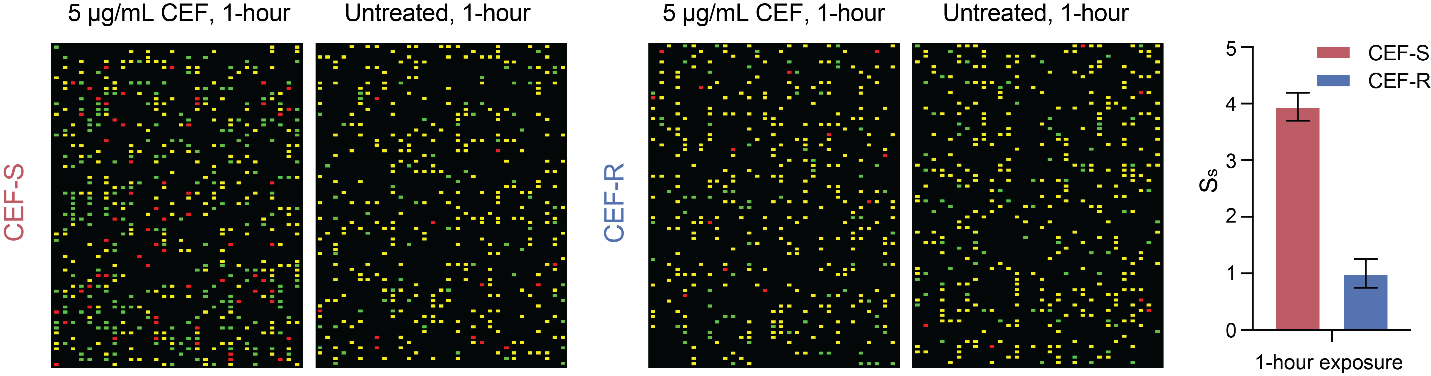


***Figure S9****.* Representative dual-digital PCR results obtained from ATCC 43069 (susceptible) and WHO-X (resistant) strains under CEF exposure at 5 µg/mL for an hour showed that they can be well differentiated with this condition. The Ss values from testing the two strains were shown in the bar plot on the right. Error bar: n=3.


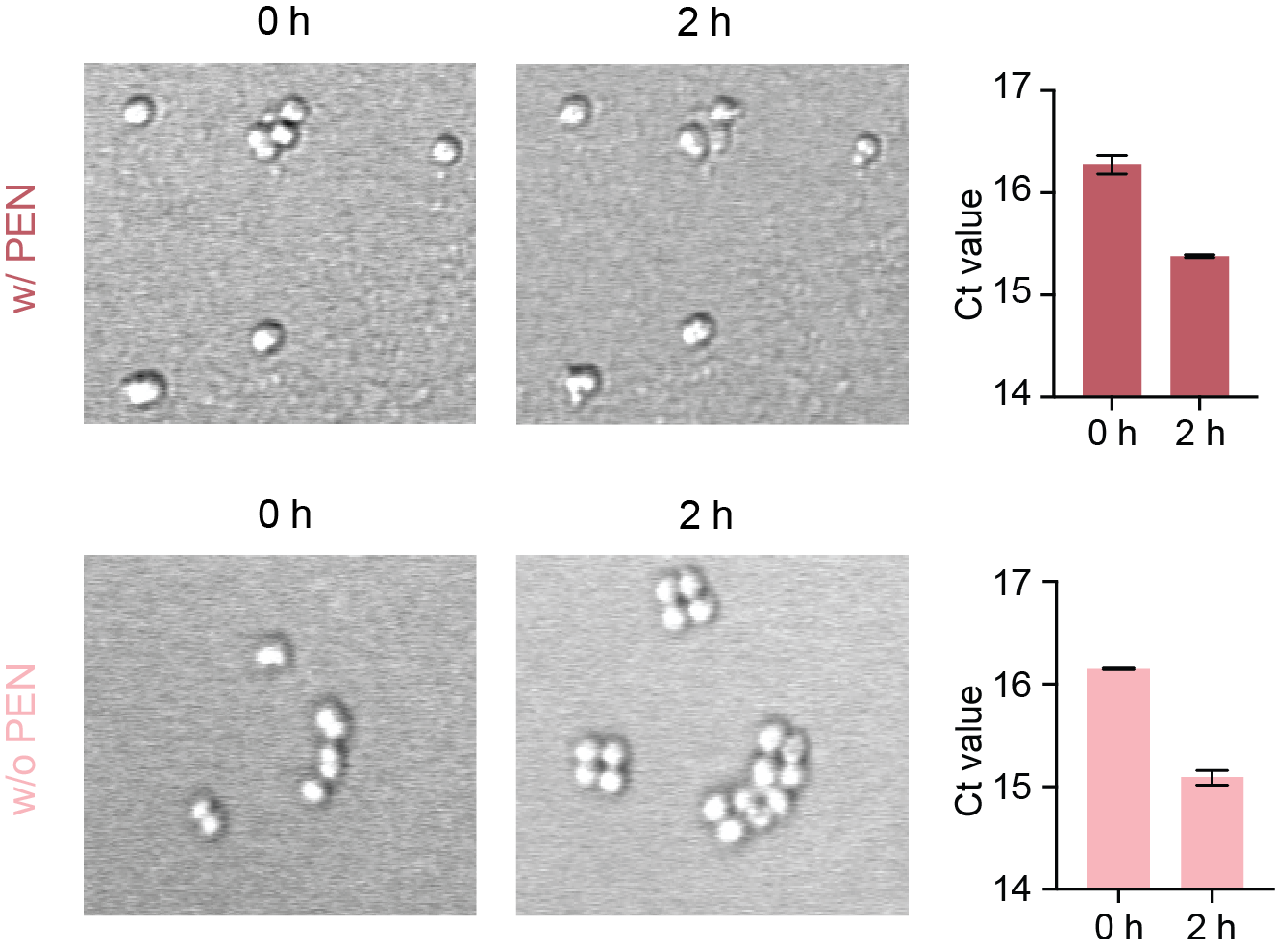


***Figure S10*.** The PEN-susceptible ATCC 43069 strain was cultured in GW medium supplemented with 2 μg/mL PEN, alongside an untreated control. Microscopic imaging of the cells was conducted, and cell suspensions were subjected to real-time PCR targeting the *opa* gene. Following a two-hour PEN exposure, while the treated group exhibited distinct bacterial count compared to the untreated group, no significant difference in Ct values was observed. This suggests that DNA replication persisted within the two-hour timeframe under antibiotic exposure. Error bar: n=3.

**
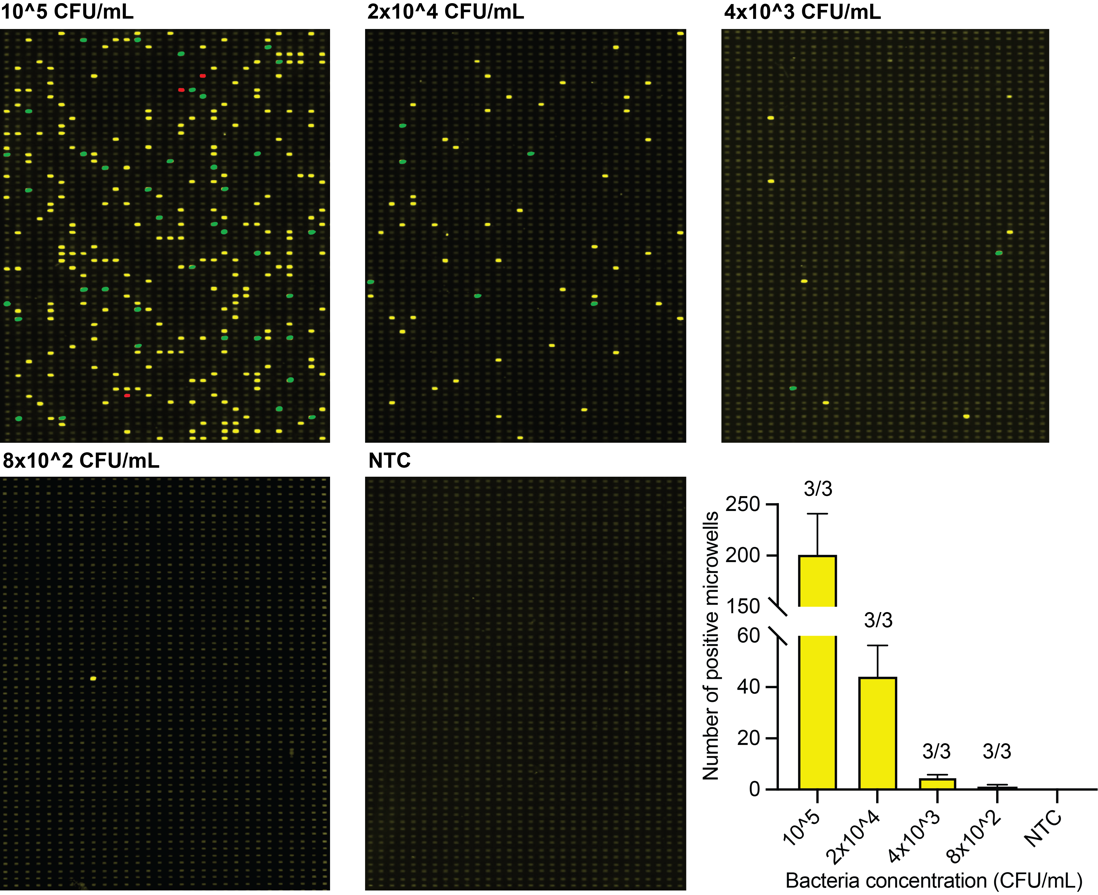
**

***Figure S11.*** Dual-digital PCR results from testing different concentrations of *N. gonorrhoeae* cell culture sample. Microwell marked in green or red represent extracellular *opa* or *terminus* DNA fragments, respectively. Microwells in yellow indicate the coincidence of *opa* and *terminus* genes, therefore being identified as intact *N. gonorrhoeae* cells for counting and evaluating the detection sensitivity of bacteria of our approach. At the lowest concentration of 8x10^2 CFU/mL, the limited number of *opa-* and *terminus-*positive microwells (one or two) was subjected to high measurement uncertainty due to sub-sampling error in digital bioassays, we therefore concluded that our current assay has a detection limit down to 4x10^3 CFU/mL. Error bars in the bar plot represent the data obtained from three replicate tests.


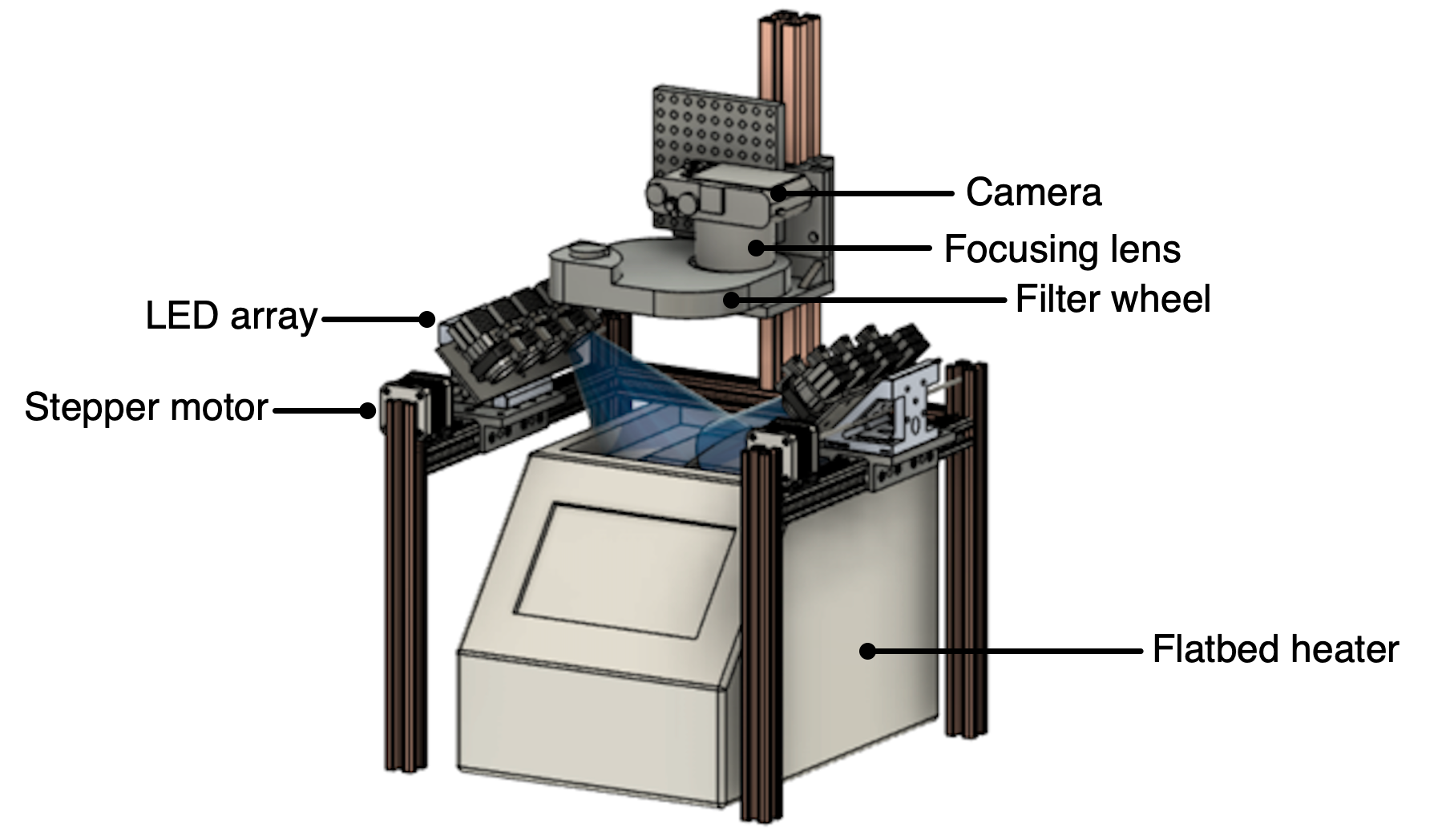


***Figure S12***. Schematic showing the structure of multi-color imaging setup.


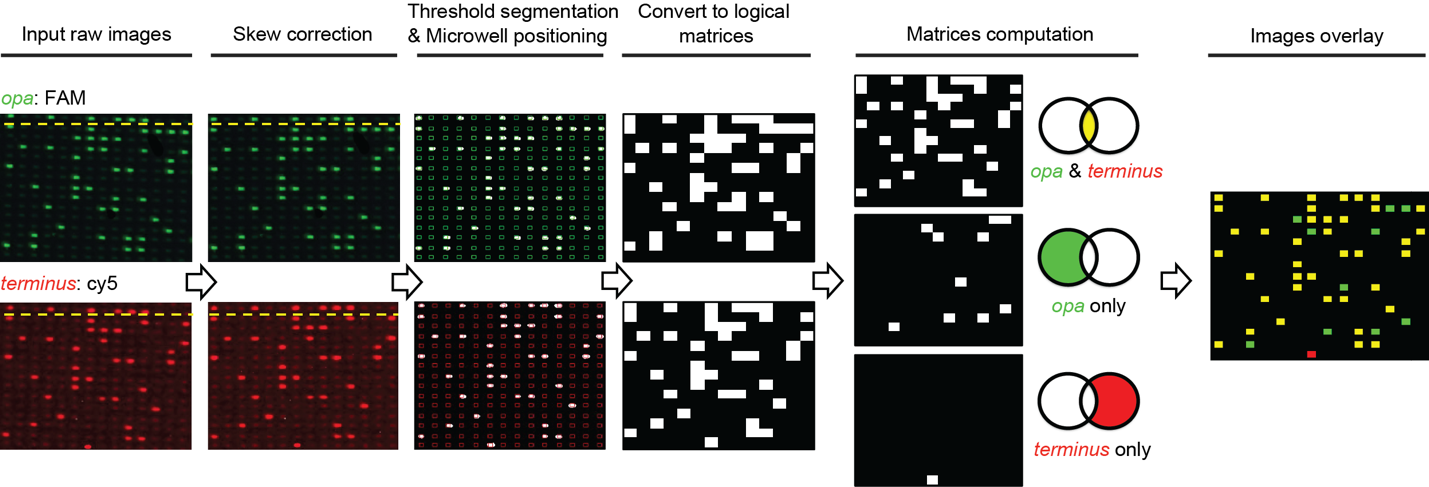


***Figure S13***. Image processing workflow for dual-digital PCR.


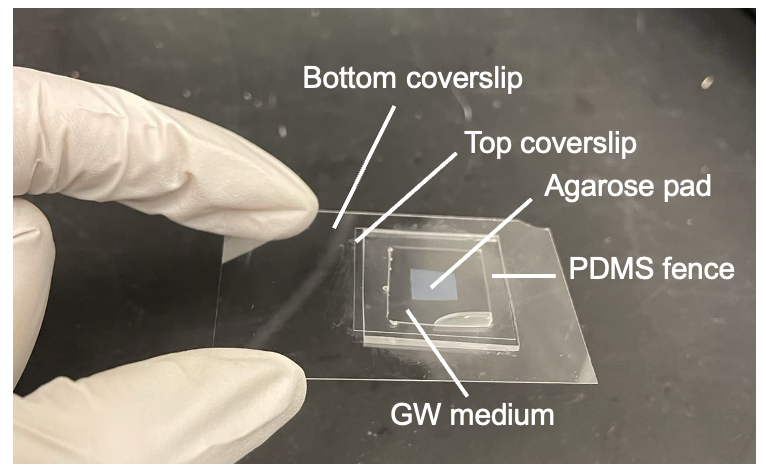


***Figure S14***. Schematic showing the structure of microliter-sized fluid chamber for bacterial culture and live-cell imaging on LSM780 microscope.

**Table S1.** E-test MIC (µg/mL) of different strains used in this paper.


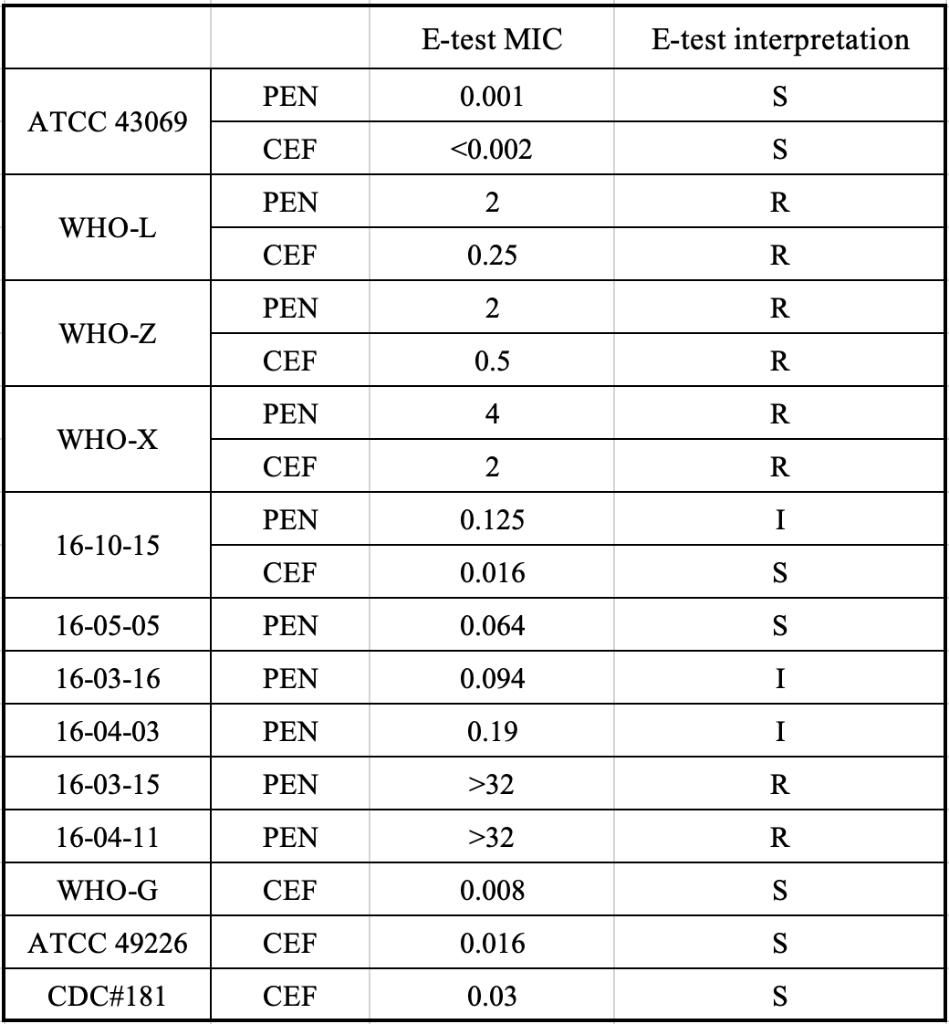


**Table S2.** Sequences of primers and TaqMan probes used in this paper.

| Name | Sequence (5’-3’) |
| --- | --- |
| Forward primer_ *opa* | TTGAAACACCGCCCGGAA |
| Reverse primer_ *opa* | TTTCGGCTCCTTATTCGGTTTAA |
| Probe_ *opa* | /56-FAM/CCGATATAA/ZEN/TCCGYCCTTCAACATCAG/3IABkFQ/ |
| Forward primer_ *terminus* | TTCCAAGGATTTGACAGAAGAAATG |
| Reverse primer_ *terminus* | GGATATGCCGCCCATACTTT |
| Probe_ *terminus* | /5Cy5/AGTTGGGCG/TAO/ATTTACTGTTTGCGC/3IAbRQSp/ |

**Supplementary Videos:**

**Video S1. Live imaging of PEN-susceptible *N. gonorrhoeae* cells under microscope.** Exposure to 2 μg/mL of PEN resulted in inhibited growth of the ATCC 43069 strain. Cells also displayed unique lysis patterns, including abrupt lysis (bottom left) and progressive lysis (top right).

**Video S2. Live imaging of PEN-resistant *N. gonorrhoeae* cells under microscope.** The clinical isolate 16-04-11, treated with 2 μg/mL of PEN, exhibited normal growth pattern during a 3-hour timeframe.

**Video S3. *N. gonorrhoeae* autolysis during cell culture in GW medium.** The untreated *N. gonorrhoeae* cells (ATCC 43069) were live-imaged under microscope. Cell that underwent autolysis was marked with yellow arrow.
